# Temperature-dependent trapping and polaron annihilation on ultrafast time scales in metal-halide perovskites

**DOI:** 10.1021/acs.jpclett.5c02164

**Published:** 2025-09-12

**Authors:** Jiacheng Wang, Jungmin Park, Lei Gao, Lucia Di Virgilio, Sheng Qu, Heejae Kim, Hai I. Wang, Li-Lin Wu, Wen Zeng, Mischa Bonn, Zefeng Ren, Jaco J. Geuchies

**Affiliations:** † State Key Laboratory of Molecular Reaction Dynamics, 58279 Dalian Institute of Chemical Physics, Chinese Academy of Sciences, 457 Zhongshan Road, Dalian 116023, P. R. China; ‡ University of Chinese Academy of Sciences, 19A Yuquan Road, Beijing 100049, P.R. China; § 28308 Max Planck Institute for Polymer Research, 55128 Mainz, Germany; ∥ Department of Physics, Pohang University of Science and Technology, 37673 Pohang, Korea; ⊥ 4496 Leiden Institute of Chemistry, Leiden University, Einsteinweg 55, 2333CC Leiden, The Netherlands; # School of Physics, Xidian University, Xi’an 710071, P. R. China

## Abstract

Understanding carrier dynamics in photoexcited metal-halide perovskites is key for optoelectronic devices such as solar cells (low carrier densities) and lasers (high carrier densities). Trapping processes at low carrier densities and many-body recombination at high densities can significantly alter the dynamics of photoexcited carriers. Combining optical-pump/THz probe and transient absorption spectroscopy we examine carrier responses over a wide density range (10^14^–10^19^ cm^–3^) and temperatures (78–315 K) in the prototypical methylammonium lead iodide perovskite. At densities below ∼10^15^ cm^–3^ (room temperature, sunlight conditions), fast carrier trapping at shallow trap states occurs within a few picoseconds. As excited carrier densities increase, trapping saturates, and the carrier response stabilizes, lasting up to hundreds of picoseconds at densities around ∼10^17^ cm^–3^. Above 10^18^ cm^–3^ a Mott transition sets in overlapping polaron wave functions leading to ultrafast annihilation, tentatively assigned as an Auger recombination process, occurring over a few picoseconds. We map out trap-dominated, direct recombination-dominated, and Mott-dominated density regimes from 78 to 315 K, ultimately enabling the construction of an electronic “phase diagram”. These findings clarify carrier behavior across operational conditions, aiding material optimization for optoelectronics operating in the low (e.g., photovoltaics) and high (e.g., laser) carrier density regimes.

## Graphical abstract

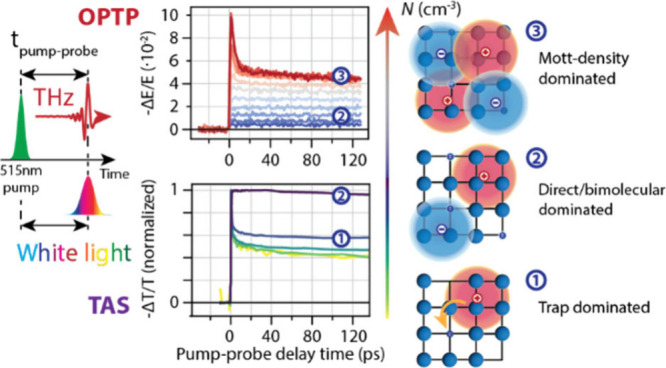

Metal-halide perovskite materials are proposed for use in various technologies as a light absorber (e.g., in photodetectors and photovoltaics) or light emitter (e.g., LEDs and lasers), each requiring distinct carrier densities. Even without external injection, methylammonium lead iodide (MAPI) intrinsically hosts charge carriers at densities below 10^12^ cm^–3^.^1^ As a solar cell material, MAPI operates at carrier densities around 10^14^ cm^–3^,^2^ where shallow traps dominate carrier dynamics.^3^ LEDs, reliant on carrier injection via electronic contacts, operate at intermediate densities around 10^12^–10^16^ cm^–3^.^4^ For MAPI to be viable for lasing technologies, carrier densities over 10^18^ cm^–3^ are required to reach population inversion.^5,6^ This wide range of carrier densities, along with the diverse applications envisioned, underscores the need to understand carrier dynamics at different carrier densities across photoexcitation fluences. In addition to hosting extra charge carriers—introduced through electrical injection, chemical doping, or photoexcitation—the perovskite lattice encompasses a multitude of defects,^7^ each with its own density,^8–17^ which can localize or trap charges.

Many reports extensively discuss various types of traps—both deep and shallow, though not always clearly defined—and their associated densities. In polycrystalline lead-halide films, reported defect densities range from 10^15^ to 10^16^ cm^–3^,^13,14,18–26^ while single crystals typically show much lower bulk defect densities, around 10^12^ cm^–3^ or lower.^13,15,22,27,28^ Recent work by Yuan et al. highlights that in both polycrystalline films and full device architectures of MAPI, shallow traps predominantly influence the time-resolved photoluminescence signals,^29^ with energy levels ranging from 50 to 130 meV away from the nearest band. Furthermore, the literature suggests the existence of distinctively different carrier density regimes where various recombination processes are predominant, occurring both at very low carrier densities^3,30^ and high carrier densities.^2,31–36^ This puts into question the concept of “intrinsic” behavior after impulsive photoexcitation of the perovskite materials.

Here, we systematically study the photoinduced carrier response at densities spanning 5 orders of magnitude (10^14^–10^19^ cm^–3^) at temperatures between 78 and 315 K in the prototypical MAPI perovskite. We use highly sensitive transient absorption spectroscopy (TAS) and optical-pump/THz probe (OPTP) spectroscopy to map the carrier dynamics after impulsive photoexcitation. In our TAS experiments, probing the carrier dynamics at 10^14^–10^15^ cm^–3^, we observe a fast decay of the photoinduced signal, caused by localization of carriers in shallow traps in several ps. Upon increasing the density to 10^16^ cm^–3^, these shallow traps become occupied, and the absorption bleach remains unchanged over hundreds of picoseconds. We extend the density range to 10^19^ cm^–3^ using OPTP, and, after an initial flat response of the time-dependent OPTP signal with increasing density, we see the onset of a fast decay over tens of ps and saturation of the carrier density, indicative of a Mott polaron transition around 10^18^ cm^–3^. We map out these carrier responses from 78 to 315 K and construct a “phase diagram”, in which we include deep trap densities from literature and a model for predicting optical gain thresholds in MAPI.

We synthesized a MAPI thin film on a water-free silica substrate using an established spin-coat-and-annealing protocol.^37,38^ The resulting polycrystalline MAPI has a preferential orientation with a [110] zone axis, as shown by room-temperature X-ray diffraction measurements (see Figure S1), and a thickness of approximately 300 nm. We sealed the film with epoxy resin in between two water-free glass substrates inside a nitrogen-purged glovebox, to prevent sample degradation under ambient conditions.^39^ Metal-halide perovskite materials host a wealth of both shallow and deep traps, which introduce levels inside the bandgap.^7,10,12,29^ Furthermore, due to the polycrystalline nature of the thin film, defects at interfaces, such as grain boundaries, cannot be avoided.^40^

To follow the evolution of the carrier dynamics as a function of photogenerated carrier density and temperature and construct an electronic “response diagram”, we combine OPTP spectroscopy and highly sensitive TAS. This combination allows us to probe the electronic response over a range of photoexcitation densities spanning 5 orders of magnitude (10^14^–10^19^ cm^–3^). Briefly, in OPTP experiments, the perovskite is first excited by a 50 fs optical pump pulse, which photoexcites electrons from the valence to the conduction band. After a controlled time delay, a single-cycle THz probe pulse, generated by optical rectification in a ZnTe(110) crystal and with an envelope duration of about 1 ps, interacts with the photoexcited electrons, which attenuates the THz field. The attenuation is a direct measure of the photoconductivity of the sample (given by the sum of products of electron and hole density and their respective mobilities). The transmitted electric field of the THz pulse is detected by an 800 nm sampling pulse via electro-optic sampling in another ZnTe(110) crystal. In TAS experiments, we used a recently developed technique based on a balanced detection scheme, allowing for an exceptional sensitivity (ΔT/T) of ∼10^–7^. This high sensitivity enabled us to probe the carrier dynamics in MAPI at carrier densities as low as 10^14^ cm^–3^. For the TAS measurements, we used a fs fiber laser (∼260 fs pulse duration) to measure the dynamics from fs to ns, and the combination of a ns diode laser and a fs fiber laser in the ns-μs time scale. We used a pump wavelength of about 515 nm in both the TAS and OPTP experiments to enable direct comparison.

For both TAS and OPTP experiments, the density *N* is inferred from the incident photon fluence and the complex refractive index at the pump wavelength^39^ (resulting in a fraction of reflected photons and a decay of the density over the absorption length, see Methods in the Supporting Information (SI)). Additionally, the photogenerated density is corrected for the photon-to-charge quantum yield, which is determined to be ∼30% in the tetragonal phase and ∼55% in the orthorhombic phase, which was determined independently from the plasma frequency obtained from fits to the THz conductivity spectra (see Figure S2). Less than unity quantum-yield in these materials is among others linked to exciton formation.^41^ As electrons and holes in MAPI have similar effective masses, both contribute nearly equally to the measured photoconductivity.

There are two types of phase transitions in MAPI over the temperature and carrier density range reported here, as shown in Figure 1(a). Around 162 K, MAPI undergoes a structural phase transition from a tetragonal structure (*I*4/*mcm*) at higher temperatures, to orthorhombic (*Pnma*) crystal structure at lower temperatures,^42,43^ shown in Figure 1(a). In addition, there are also distinct electronic “phases”, depending on the carrier density: at high densities, where polaron wave functions start to overlap, there is a Mott transition in which polarons rapidly annihilate over tens of picoseconds.^33,36^ Here, we measured TAS at carrier densities from 3 × 10^14^ to 4 × 10^16^ cm^–3^, and OPTP from 5 × 10^16^ to 1 × 10^19^ cm^–3^.

**Figure 1:**
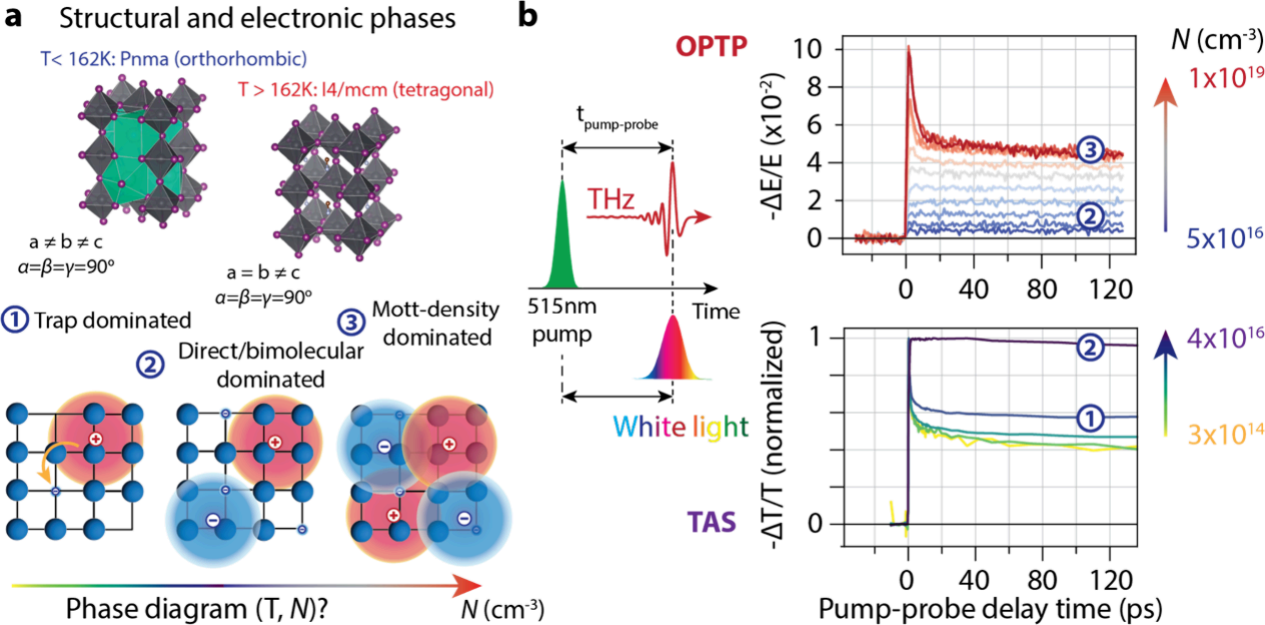
Structural and electronic phases in methylammonium lead iodide. (a) MAPI adopts two crystal structures over the temperature range we have investigated; above 162 K it is in the tetragonal (*I*4/*mcm*) phase, whereas below it is in the orthorhombic crystal phase. Furthermore, we will show throughout the paper that the dynamics of photoexcited carriers are dominated by (1) trap-assisted recombination (up to 10^15^ cm^–3^), (2) direct bimolecular recombination (10^15^–10^18^ cm^–3^), and (3) above the Mott density (>10^18^ cm^–3^), fast polaron annihilation. It is important to note that the trap density is not a material-specific property, but depends on the sample quality, whereas the Mott density is an intrinsic material property. (b) The combination of THz and TA pump–probe spectroscopy allows us to probe photoexcited electrons in perovskites over a range of densities spanning 5 orders of magnitude. THz photons serve as a probe for higher densities (5 × 10^16^–1 × 10^19^ cm^–3^), whereas a white-light probe pulse in transient absorption spectroscopy was used as a probe at low densities (3 × 10^14^–4 × 10^16^ cm^–3^). The electronic phases from panel (a) are indicated in the pump–probe transients.

Figure 1(b) shows an example of data recorded at a temperature of 100 K after photoexcitation at 515 nm. The bottom panel shows TAS data in the low-to-medium density range [range 1 and 2 in Figure 1(a)]. At low carrier densities, monomolecular recombination (trap-assisted recombination) dominates carrier recombination. In addition, there is a fast decay over a few ps of the band-edge bleach signal, which we will show indicates the loss of either electrons or holes to shallow trap states. As the carrier density is increased, this fast decay disappears, and the band-edge bleach becomes constant over the first 130 ps, indicating that the fraction of trapped carriers relative to the total photoexcited carrier density decreases and becomes negligible. The decay, mainly through a bimolecular recombination process, and the hot phonon bottleneck effect act together, causing the bleaching signal to remain almost unchanged over the first hundred picoseconds. The top panel shows OPTP data in the medium-to-high carrier density range. Initially, at lower pump fluences, the signal is constant over the first 140 ps. As we increase the pump fluence, the instantaneous photoconductivity increases, but decays fast over the first 10 ps to a constant value, to a density corresponding to the Mott density, which will be discussed later. We start the discussion at low photoexcited carrier densities.

In the TAS measurements, we initially performed spectral measurements at varying temperatures to characterize the ground-state bleach (GSB) feature, see Figure S3. The spectral position of the GSB changes across the tetragonal-to-orthorhombic phase transition in MAPI at 160 K. Figure 2 shows the temperature- and carrier density-dependent TAS measurements at 120 (a), 200 (b), and 291 K (c). The data for all temperatures can be found in Figure S4. Reducing the photoexcited carrier density (to ∼10^14^–10^15^ cm^–3^) causes the normalized TA curves to converge at all temperatures. The nature of trapping is such that a maximum fraction of charge carriers recombine at low carrier densities.^3^ We captured this quantitatively by plotting the ratio of ΔT/T at 10 ps, *B*, after the initial fast decay, over the instantaneous ΔT/T, *A*, which are shown in Figures 2(d–f) and Figure S5. Saturation of the B/A ratio at low carrier densities indicates entry into the linear response region, where trap-assisted recombination dominates, lasting tens of nanoseconds, and direct electron–hole recombination and Auger processes are negligible. This region mirrors the intrinsic carrier dynamics of any particular perovskite material with a given trap density under solar illumination (∼10^14^ cm^–3^).^3,30^ Across various sample types and preparation methods, a shallow-trapping fraction of tens of percent of the carriers at low temperatures and low carrier densities is typical and seemingly inevitable. Note that the maximum carrier density to enter the linear region depends on temperature, decreasing from 4.3 × 10^15^ cm^–3^ at 291 K to 4.3 × 10^14^ cm^–3^ at 120 K, but the general trend remains consistent.

**Figure 2:**
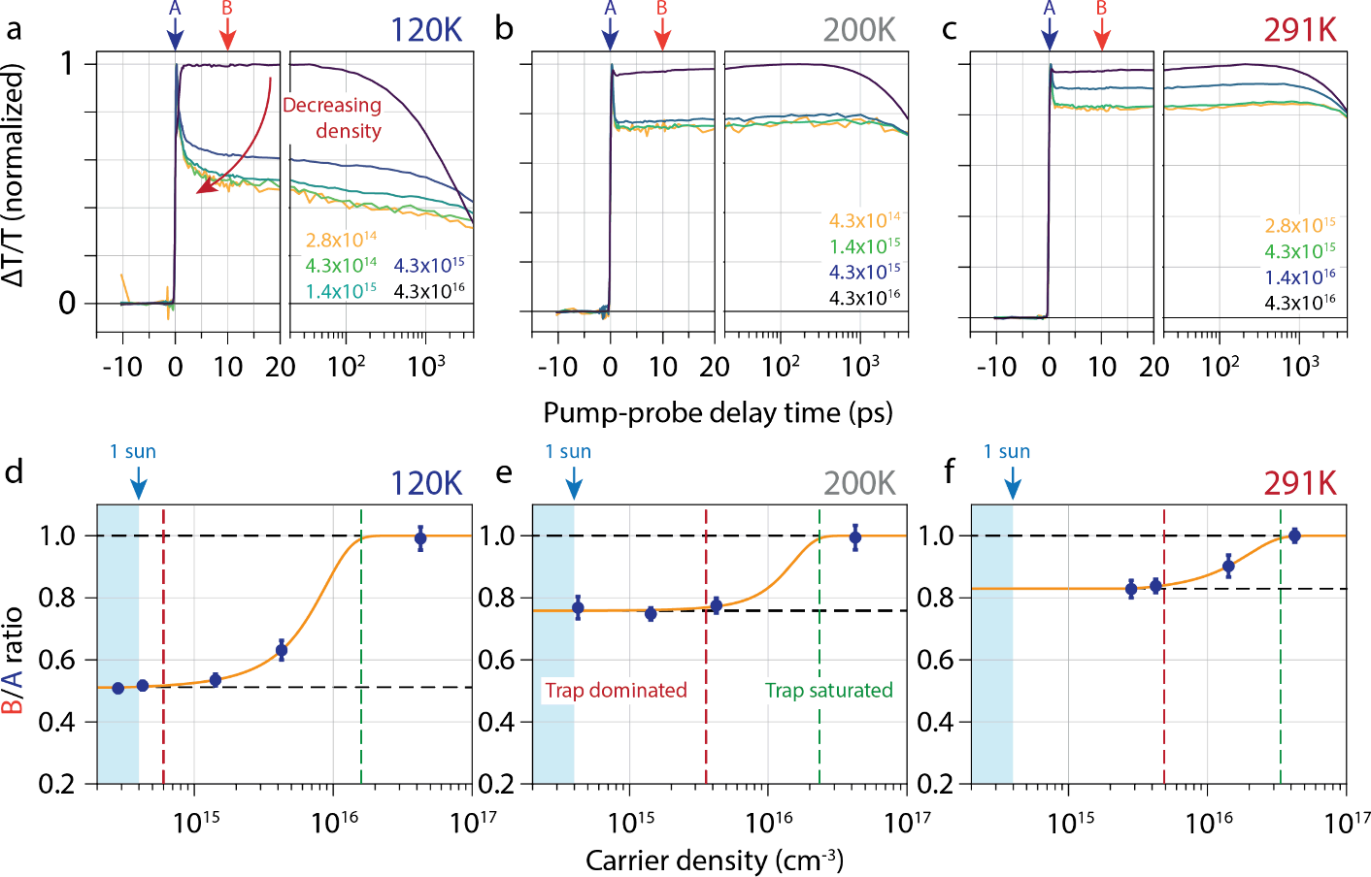
Temperature- and carrier-density-dependent TAS dynamics at different temperatures. (a–c) Density-dependent TA traces at *N* < 10^16^ cm^–3^ at 120 K (a), 200 K (b) and 291 K (c). At low carrier densities, there is a fast decay of the ΔT/T signal, which we attribute to fast carrier localization into shallow trap states, which get saturated at higher carrier densities. Note that we reach a true linear photoinduced response at the lowest carrier densities at each temperature. The corresponding carrier densities are indicated in the figures. (d–f) Ratio of ΔT/T at 10 ps, *B*, divided by the instantaneous ΔT, *A*, as a function of carrier density at (d) 120 K, (e) 200 K and (f) 291 K. The carrier densities at which we observe a true linear response are indicated by vertical red lines and the carrier densities at which the fast decay vanishes are indicated by vertical green lines. The yellow lines are fits of the data points to an error function.

The reduction of the GSB in the linear response range, which displays a rapid decay within a few picoseconds, is attributed to the trapping of carriers into shallow trap states.^3^ As the temperature decreases, a greater proportion of carriers become trapped, because the reduced thermal energy limits their ability to detrap from shallow defect levels. Traps that are shallow at high temperature can act as deep traps at low temperatures, which is also reflected by the decreasing amplitude of the B/A ratio as a function of carrier density for increasing temperatures (see Figure S6). This results from the balance between rapid trapping and reduced thermally activated detrapping. As the pump fluence is increased, the relative amplitude of this initial fast decay becomes vanishingly small, indicating that the fraction of trapped carriers relative to the total photoexcited carrier density decreases, and can even be considered negligible, as these shallow trap states become saturated. At higher carrier densities, 10^16^–10^17^ cm^–3^, the carrier dynamics are dominated by direct or bimolecular recombination. The decay, mainly through a bimolecular recombination process, has a longer lifetime, causing the bleaching signal to remain almost unchanged over hundreds of picoseconds (see Figure S7).

As shown in Figures S8–S9 and Table S2, the carrier lifetime at 100 and 120 K extends over several hundred nanoseconds, whereas at higher temperatures, it lasts about tens of nanoseconds. Interestingly, the carrier lifetimes of the orthorhombic structure at low temperatures (100 and 120 K) are nearly identical. Similarly, the carrier lifetimes of the tetragonal structure at high temperatures (170, 200, 220, 250, 270, and 291 K) are also similar, suggesting that the lifetimes are primarily crystal-phase-dependent. Note that in our TAS experiments, we use pump and probe lateral sizes of three and one mm respectively (see experimental methods). Effects such as drift and diffusion do not occur over these length- and at time scales.^29^ Factors affecting carrier dynamics, in addition to crystal structure, may also include the energy- and spatial distribution of shallow- and deep-level defect states.

We next discuss the carrier dynamics in the medium-to-high carrier density range from 10^16^ to 10^19^ cm^–3^. Similar to our earlier work,^33,36^ various features appear in the OPTP transients at low temperatures shown in Figure 3(a): at low carrier densities (*N* < 10^18^ cm^–3^), the amplitude of the photoconductivity increases linearly with excitation density and shows a modest decay over the pump–probe delay window in our experimental setup (∼1 ns, see ). The low density ensures that the overlap between polaron wave functions is small and there is limited fast bimolecular recombination (i.e., polaron–polaron annihilation). At higher densities, *N* > 10^18^ cm^–3^, the peak photoconductivity increases sublinearly with *N*, and decays rapidly within the first tens of ps to a constant level. This behavior is summarized by plotting the peak photoconductivity (blue data points) and the photoconductivity at late pump–probe delay times (200 ps, yellow data points) in the inset of Figure 3(a), which reveals the presence of a critical density, *N*_Mott_. At excitation densities exceeding *N*_Mott_, the peak photoconductivity increases sublinearly with *N*, while the photoconductivity at later times reaches a plateau. To accurately determine this critical density, we extrapolate the photoconductivity signal from OPTP traces at high carrier densities at late times back to a pump–probe delay of zero, as indicated by the black dashed line. We utilize the linear relationship established for the photoconductivity at low pump fluences to determine *N*_Mott_, which is found to be (9.02 ± 0.06) × 10^17^ cm^–3^ at 120 K. Similar behavior is observed at temperatures above 162 K, as illustrated in Figures 3(b) and 3(c). In these cases, where MAPI adopts a tetragonal crystal structure, the photoconductivity exhibits minimal decay at densities below 5 × 10^17^ cm^–3^. At higher densities, a rapid decay occurs within the first tens of picoseconds, followed by a saturation of the photoconductivity above *N*_Mott_. The extracted Mott densities are (1.86 ± 0.08) × 10^18^ cm^–3^ and (2.9 ± 0.2) × 10^18^ cm^–3^, at 200 and 292 K, respectively. This is consistent with the formation of large polarons, which screen carriers from defects and other charge carriers,^44–49^ as we will show below.

**Figure 3:**
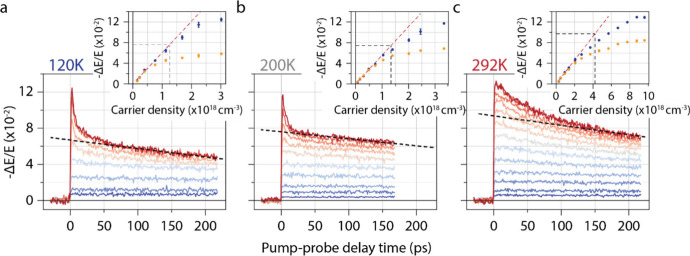
Temperature- and carrier density-dependent polaron decay dynamics measured in the carrier density range of 10^16^–10^19^ cm^–3^ with OPTP. (a–c) Density dependent OPTP traces at *N* > 10^16^ cm^–3^ at (a) 120 K, (b) 200 K and (c) 292 K. As the photogenerated density increases, the polaron wave functions start to overlap and they annihilate until the density reaches the Mott density. The insets show the instantaneous −ΔE/E signal (blue data points) and late-time −ΔE/E (yellow data points), which are used to determine the Mott density (black dashed lines). The red dashed line is a linear fit through the low-fluence data.

Combining the results from both TAS and OPTP over a wide temperature range allowed us to construct “phase diagram” for the electronic response presented in Figure 4. This diagram categorizes the data across the temperature spectrum, distinguishing between the tetragonal and orthorhombic phases of MAPI above and below 160 K, respectively. At densities <10^15^ cm^–3^, fast carrier localization into shallow trap states dominates the photoinduced response. We have extracted the true linear-reponse density, where trap-assisted recombination is dominant, and the shallow-trap-saturated density from the TAS measurements, which are shown as red and green data points, respectively. Direct recombination dominates for intermediate densities spanning 10^15^–10^17^ cm^–3^, and photoexcited electrons and holes recombine over a time scale exceeding 1 ns. At densities above 10^18^ cm^–3^, the Mott polaron density, fast polaron–polaron annihilation (likely via an Auger-type mechanism) occurs over a few tens of ps, eventually stabilizing at the Mott density. The Mott densities, obtained by OPTP, are shown as blue data points and vary from (1.34 ± 0.06) × 10^18^ cm^–3^ at 78K to (4.5 ± 0.1) × 10^18^ cm^–3^ at 315 K. We can use these obtained Mott densities to estimate the polaron radii as a function of temperature (Figure S12). The polaron radius decreases from approximately 6.5 nm at 78 K to about 4 nm at 315 K. This trend suggests the formation of large polarons that extend over multiple unit cells, in line with Feynman’s polaron theory.^33,36,47,48,50^ The depth profile of the density of deep trap levels in MAPI, was determined by Ni et al.,^13^ and varies from 1.8 × 10^11^ cm^–3^ in the bulk to 3.1 × 10^12^ cm^–3^ at the surface and interfaces in MAPI (shown by the purple shaded area).

**Figure 4:**
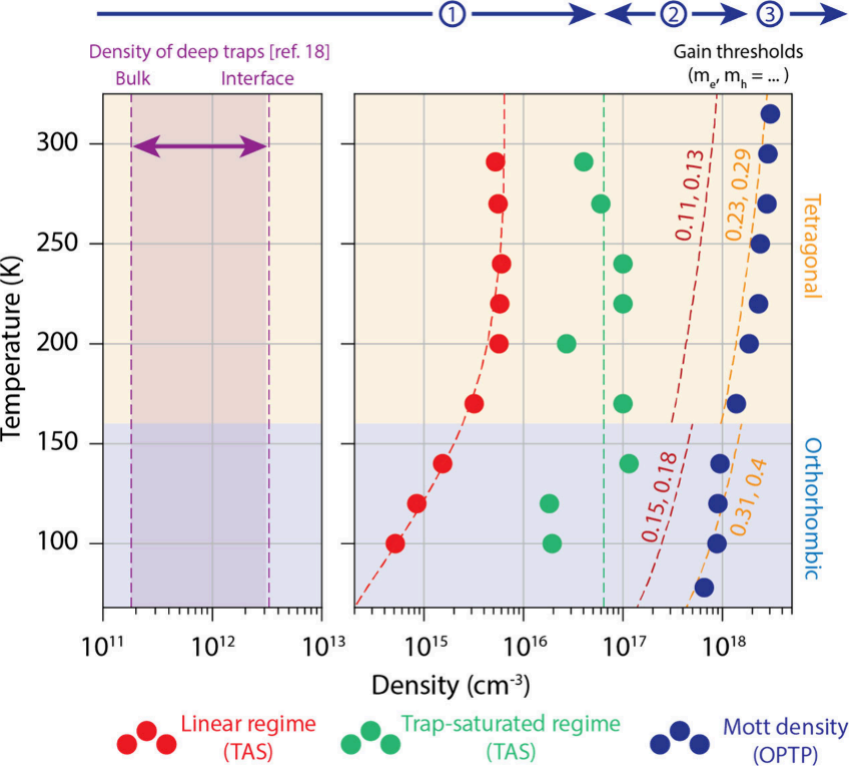
Temperature-dependent electronic “phase diagram” of MAPI spanning 8 orders of magnitude in carrier density. The electronic “phase diagram”, was obtained by combining the TAS and OPTP data: the carrier densities corresponding to the linear- and trap-saturated regime were obtained by TAS, the density of deep traps at the interface and the bulk are taken from ref 12, the Mott density is obtained from OPTP spectroscopy. Gain thresholds for various effective masses in the tetragonal and orthorhombic crystal phases are calculated as explained in the text. The three numbered ranges at the top refer to the electronic density regimes displayed in Figure 1(a).

These results demonstrate that distinct carrier dynamics can be observed at different photogenerated carrier densities, and also emphasize the importance of carefully reporting the carrier densities before concluding the nature of rapidly decaying signals and ascribing them to exact physical effects. This is illustrated by the similar transient signatures of polaron–polaron annihilation at high densities, and fast trapping of carriers into shallow defect states at low densities, both occurring on the few-to-tens of ps time scales but which have opposite dependencies on carrier density (i.e., incoming photon fluence).

It is crucial to note that *trap densities are indicative of material quality*, and hence depend on the specific preparation conditions of perovskite thin films. In contrast, the *Mott density is an intrinsic property of the material*, which remains constant regardless of the synthesis details but can be altered by changing the crystal structure or chemical composition.

A possible application for perovskite-based materials at high carrier densities is as a gain medium for lasers. Amplified stimulated emission and lasing have been demonstrated in thin-film MAPI,^5,51,52^ but concerns have also been raised over data interpretation.^53^ In order to reach population inversion, the first requirement for light amplification, the difference in quasi-Fermi-level-splitting for electrons and holes has to be larger than the bandgap.^5,54–56^ Using the effective masses for the electrons and holes (m_e_ = 0.11 m_0_, m_h_ = 0.13 m_0_, with m_0_ the rest mass of an electron)^57,58^ (see SI for the full model description), we estimate that a carrier density of 8 × 10^17^ cm^–3^ is required for population inversion at room temperature, slightly below the experimentally observed Mott density of (4.2 ± 0.2) × 10^18^ cm^–3^. Over the entire range of experimentally measured temperatures, indicated by orange dashed lines in Figure 4, we observe a notable jump in gain thresholds around 162 K, coinciding with the structural phase transition. In the orthorhombic phase, the carrier effective mass is roughly 36% higher (m_e_ = 0.15 m_0_, m_h_ = 0.18 m_0_).^59–61^ This increase in effective masses leads to a higher electronic density of states (DOS), necessitating a greater density of photoexcited carriers to achieve population inversion. However, a recent study using angle-resolved photoemission spectroscopy has determined even larger effective masses for electrons and holes: m_e_, m_h_ = 0.23 m_0_, 0.29 m_0_.^62^ The resulting gain thresholds vs temperature, shown as orange dashed lines, have been scaled in the orthorhombic phase of MAPI using the same factor applied to other reported effective masses. Interestingly, these gain thresholds align closely with the corresponding Mott densities.

Note that across the entire temperature range, using these effective masses, the density required for population inversion aligns with or is below the Mott density. This is encouraging for the development of perovskite materials as a gain medium for lasing applications. Additionally, effects such as bandgap renormalization and strong electron–phonon interactions—both prevalent in MAPI^63,64^ —can further lower the estimated gain threshold. These interactions can shift stimulated emission out of the absorbing part of the spectrum, enhancing the material’s lasing potential.

By combining ultrasensitive transient absorption and optical-pump/THz probe spectroscopy, we have captured the ultrafast response of photoexcited carriers in the prototypical perovskite methylammonium lead iodide across carrier densities ranging from 10^14^ to 10^19^ cm^–3^. A comprehensive analysis allowed us to construct an electronic ‘phase diagram’ for temperatures ranging from 78 to 315 K. Our findings reveal that at densities below ∼10^15^ cm^–3^, there is fast trapping of photoexcited carriers within one-to-several ps into shallow traps, revealed by a rapid decay of the TAS signals. At densities up to 10^18^ cm^–3^, the Mott density, the ‘shallow-trap-saturated regime’ is probed in which the dynamics are constant over the first ns and increase linearly with fluence. Above the Mott density, polaron wave functions begin to overlap, causing rapid annihilation of photoexcited polarons within tens of picoseconds, and their size shows the formation of large polarons, consistent with the temperature dependence predicted by Feymann’s polaron theory. We demonstrate that the Mott densities exceed both experimentally determined and predicted thresholds for light amplification, underscoring the potential of MAPI as a gain medium. Accurate determination of photoexcited carrier densities is important when reporting ultrafast spectroscopic data, not just for perovskite-based materials, as the transient signals of excited electrons can vary, and may even reverse its dependence on excitation fluence on picosecond time scales, depending on the density range that is probed experimentally.

## Supplementary Material





## Notes

Sample preparation and additional steady-state characterization, description of the THz and TAS spectroscopic setups, additional TAS and OPTP data, and modeling the threshold for optical gain (PDF)
Transparent Peer Review report available (PDF)
