# Supplementary material for: Temperature-dependent trapping and polaron annihilation on ultrafast time scales in metal-halide perovskites
Source: J Phys Chem Lett. 2025 Sep 12;16(38):9925–32. doi: 10.1021/acs.jpclett.5c02164 (PMC12478862; doi:10.1021/acs.jpclett.5c02164)
Supplement: Supplementary file 1 [file jz5c02164_si_001.pdf]

Supporting information

Temperature-dependent trapping and polaron annihilation on ultrafast timescales in metal-halide perovskites

Jiacheng Wang[#,1,2], Jungmin Park[3], Lei Gao[3], Lucia Di Virgilio[3], Sheng Qu[3], Heejae Kim[3,4], Hai I. Wang[3], Li-Lin Wu[1,6], Wen Zeng[1,2], Mischa Bonn[3*], Zefeng Ren[*,1,2], Jaco J. Geuchies[3,5#*]

1. State Key Laboratory of Molecular Reaction Dynamics, Dalian Institute of Chemical Physics, Chinese Academy of Sciences, 457 Zhongshan Road, Dalian 116023, P. R. China

2. University of Chinese Academy of Sciences, 19A Yuquan Road, Beijing 100049, P.R. China

3. Max Planck Institute for Polymer Research, 55128 Mainz, Germany.

4. Department of Physics, Pohang University of Science and Technology, 37673, Pohang, Korea.

5. Leiden Institute of Chemistry, Leiden University, Einsteinweg 55, 2333CC, Leiden, the Netherlands.

6. School of Physics, Xidian University, Xi'an 710071, P. R. China

Table of Contents

SAMPLE PREPARATION 3
ULTRAFAST SPECTROSCOPIC METHODS 4
Optical-pump/THz probe spectroscopy *4*
Highly-sensitive transient-absorption spectroscopy *5*
Determination of the photoexcitation density *6*
Photoconductivity spectra and photon-to-carrier quantum yield *8*
Full TAS dataset and analysis *10*
Full OPTP dataset and analysis *16*
Estimation of the temperature-dependent polaron size *20*
Modeling the threshold for optical gain/population inversion *22*
SUPPLEMENTARY REFERENCES 24

Sample preparation

Methylammonium iodide (MAI) was purchased from Greatcell Energy. Lead acetate [($Pb(Ac)_2$) 98% purity] was purchased from Tokyo Chemical Industry. Anhydrous dimethylformamide (>99.8% purity) was purchased from Sigma Aldrich. Substrates (0.3mm-thick glass) were cleaned by sonication in water with soap for 30 minutes, afterwards rinsed with deionized water three times, and again sonicated in ethanol and acetone (both for 30 minutes). Afterwards, the substrates were dried under a nitrogen flow and subjected to UV-Ozone treatment (FHR UVOH 150 LAB, 250 W) for 20 minutes with an oxygen feeding rate of 1L/min right before spincoating. Film preparation was carried out in a nitrogen purged glovebox. The precursor solution consists out of 477mg (3mmol) MAI and 325.3mg of $Pb(Ac)_2$ (1mmol) in 1 mL DMF. The film preparation was done by depositing 50 μL of the precursor solution onto a cleaned fused silica substrate and spincoating it at 4000 rpm (ramp ± 1000 rpm/s) for one minute. Afterwards the film was left to dry at room temperature for 5 minutes and subsequently annealed on a 100°C hotplate. We have sealed the film with epoxy-resin in-between two water-free glass substrates inside a nitrogen purged glovebox, to prevent sample degradation under ambient conditions.

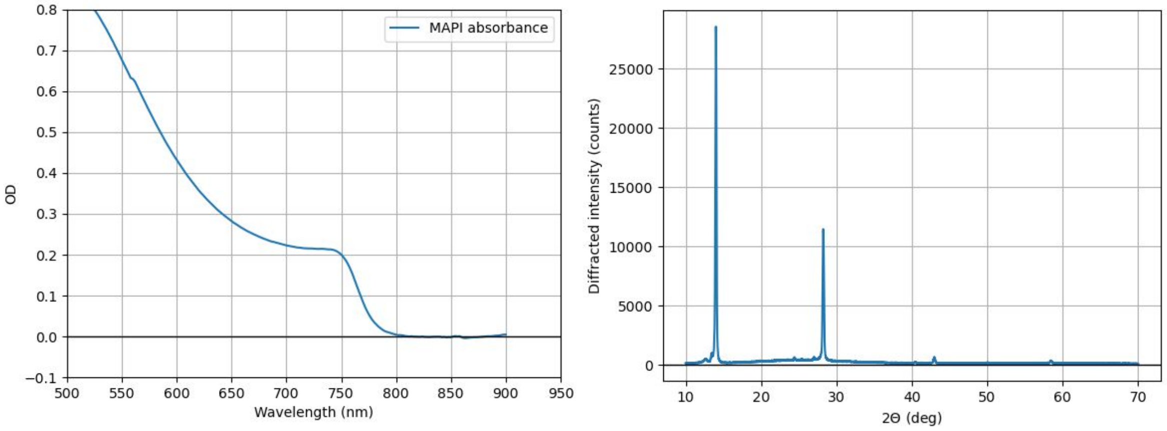

Figure S1: Absorption spectrum (left) and powder X-ray diffraction pattern of the [110] oriented MAPI film used throughout this work. The absorption spectrum was measured by placing the sample inside an integrating sphere. X-ray diffraction was performed on a Rigaku Smartlab diffractometer, using copper Kα radiation (1.54 angstrom) in Bragg-Brentano geometry.

Ultrafast spectroscopic methods

Optical-pump/THz probe spectroscopy

Here, we describe the technique THz time-domain spectroscopy (TDS) and Optical Pump THz Probe Spectroscopy (OPTP). This technique can probe the photoconductivity of carriers and further retrieve the charge carrier mobility μ.

We use an amplified Ti:sapphire laser (Spitfire Ace, Spectra-Physics) producing pulses with 800 nm central wavelength and ~50 fs pulse duration at 1 kHz repetition rate. The THz field is generated by optical rectification in a ZnTe(110) crystal (thickness 1 mm). The THz detection is based on the electro-optic (also called Pockels) effect in a second ZnTe crystal with 1 mm thickness. We vary the time delay between the THz field and

the 800 nm sampling beam with a motorized delay stage (M-605.2DD purchased from Physik Instrument (PI)). The time delay between the optical pump and THz probe pulses is controlled by a second motorized delay stage (M521.DD, Physik Instrument (PI)). The pump-pulse is generated in an optical parametric amplifier (OPA) to convert our 800nm fundamental beam into 515 nm, to match the experimental conditions between the OPTP and TAS measurements, which we filter further using a 515±10 nm bandpass filter (FBH515-10, Thorlabs) directly after the OPA.

Highly-sensitive transient-absorption spectroscopy

The transient absorption measurements were based on a fiber laser (1035 nm, pulse duration of ~230 fs, 1 MHz repetition rate). The sensitivity level ($\Delta T/T$) of 10^{-7} was achieved by a novel technique of combining macropulse and micropulse and using a balanced detector scheme. The output was split into two beams. The first beam (pump) frequency doubled by second harmonic generation. The pump light was modulated at 500 Hz of macropulses by a chopper. The other beam of 1035 nm was focused into a sapphire crystal to generate a broad supercontinuum probe pulse. Afterwards, it was split into two beams by using an achromatic waveplate and a pellicle beam splitter (PBS). One was guided over a monochromator and was collected by a photodiode (PD) as the reference light, and the other was focused and then passed through the sample as the probe light, collimated and dispersed with a monochromator, and finally probed by a second PD, with an integrating 3 nm bandwidth (FWHM) of the probe light. Both

PDs were connected to a balanced transimpedance amplifier and then a lock-in amplifier. For the TA measurements at longer pump-probe delay times, a diode laser (516 nm, FWHM 2.2 nm, NPL52C, Thorlabs) was used as the pump, which was synchronized with the fiber laser. Their delay was electronically tuned with a delay generator.

Determination of the photoexcitation density

The photon density impinging on the sample can be calculated as

$$n_{photon} = \frac{E\lambda}{hc} = \frac{Power\ (W)}{repetition\ rate\ (s^{-1}) \cdot \frac{h \cdot c}{\lambda}\ (J)\ \cdot \pi r^2\ (cm^2)}$$

A fraction of the incoming light is reflected, F_R, at the perovskite-air interface, which can be calculated using the following equations

$$F_A \cdot F_R = (1 - 10^A) \cdot F_R = (1 - 10^A) \cdot \left|\frac{n_1 - n_2}{n_1 + n_2}\right|^2$$

where n_1 and n_2 are the refractive indexes of the materials at the pump wavelength (515 nm), A is the absorption value at the corresponding wavelength. Here, $n_1 = 1 + 0i$ (for air) and $n_2 = 2.9992 + 0.3523i$ (for $MAPbI_3$)[1]. Since the thickness of the film (~300 nm) is much thicker than the absorption length at the pump wavelength ($L_{abs,\,515nm} = \frac{1}{\alpha} = \frac{\lambda}{4\pi\,\kappa} = \frac{515\ nm}{4\pi\ 0.3523} \approx 116\ nm$), we set $F_A = 1$. To calculate the photoexcited carrier density, we have to take into account that the pump beam

attenuates over the absorption length in the sample following the Beer-Lambert law, characterized by a decay length of a few tens nanometers from the surface, as

$$I(x) = I_0 e^{-\alpha x}$$

The corresponding absorbed light flux over the absorption length/penetration depth is

$$\left(\frac{1 - e^{-\alpha L}}{\alpha L}\right) \cdot n_{photon}$$

where α is the absorption coefficient, L (= 1/ α) is the penetration depth at the pump wavelength (or the sample thickness if this is smaller than the penetration depth).

Considering all the parameters, the modified absorbed carrier density can be expressed as:

$$N_{abs} = \frac{Power}{repetition\, rate \cdot \frac{h \cdot c}{\lambda} \cdot \pi r^2 \cdot L} \cdot (1 - 10^A) \cdot \left|\frac{n_1 - n_2}{n_1 + n_2}\right|^2 \cdot \left(\frac{1 - e^{-\alpha L}}{\alpha L}\right)$$

The photogenerated carrier density N in THz spectroscopy (not for TAS) is inferred from the absorbed carrier density, and the photon-to-carrier quantum yield Φ: $N = \Phi N_{abs}$. The absorbance of MAPI at 515 nm is nearly temperature-independent[2], and hence we have used the room temperature refractive index at this wavelength for the calculations of the photoexcited carrier density.

Photoconductivity spectra and photon-to-carrier quantum yield

We have determined the photo-to-carrier quantum yield Φ independently, by recording a photoconductivity spectrum at low fluence (and hence carrier density) for the MAPI film we have measured. This was fitted with the Drude-Smith model, in order to extract the plasma frequency, proportional to the carrier density:

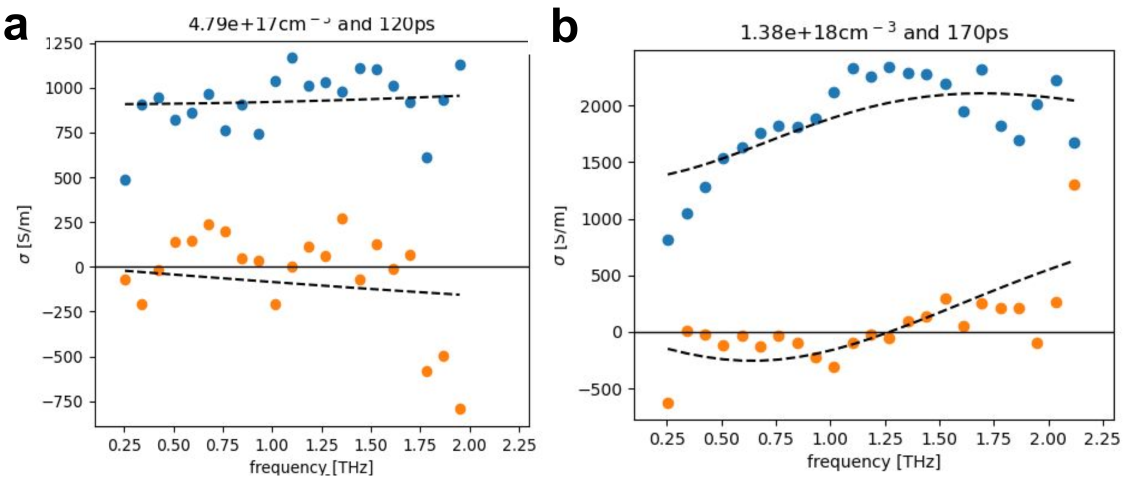

Figure S2: Photoconductivity spectra at low photoexcited carrier densities and late pump-probe delay times at (a) 78 K and (b) 292 K. Blue and orange datapoints correspond to the real and the imaginary part of the complex photoconductivity respectively. The dashed lines are a fit to the Drude-Smith model.

$$\Delta\tilde{\sigma}(\omega) = \frac{\varepsilon_0 \omega_p^2 \tau_0}{(1 - i\omega\tau_0)} \cdot (1 + \frac{C}{1 - i\omega\tau_0})$$

ω_p is the plasma frequency, ε_0 is the vacuum permittivity τ_0 is the elastic scattering time, and C is the backscattering constant, a measure for the probability that the charge maintains its initial velocity during scattering ranging from 0 (Drude scattering only, represents full momentum randomized scattering) to -1 (backscattering only). From the fit, the charge carrier density ($N = \omega_p^2 m^* \frac{\varepsilon_0}{e^2}$) can be extracted and the photon-to-carrier quantum yield Φ can be calculated by comparison with the absorbed photon density. The results of the Drude-Smith fits to the photoconductivity spectra are shown in the table below.

**Temperature**	$\boldsymbol{\tau_0}$ **(fs)**	$\boldsymbol{\omega_p}$ **(THz)**	$\boldsymbol{C}$
78 K	53±10	63±7	-0.55±0.05
292 K	72±4	81±3	-0.67±0.02

Supplementary table 1: Results from the fits of the Drude-Smith model to the photoconductivity spectra shown in Figure S2.

We obtained photon-to-carrier quantum yields of 0.55 at 78 K (for the orthorhombic phase), and 0.3 at 292 K (for the tetragonal phase).

Full TAS dataset and analysis

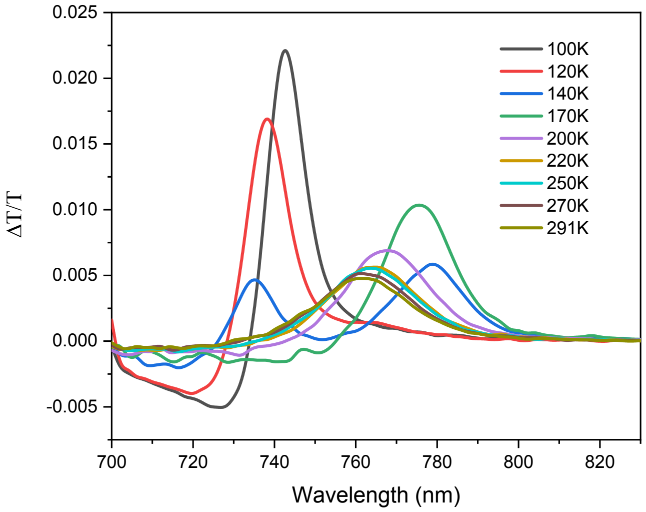

Figure S3: TA spectra at different temperatures. Ensemble of TA spectra of $MAPbI_3$ thin films at different temperatures and changes of transmission ΔT/T were plotted at a pump-probe delay of 1 ns. The pump energy was 10 nJ per pulse with a diameter of 3.5 mm and a wavelength of 517.5 nm. Note that two GSB peaks imply the presence of two phases at 140 K.

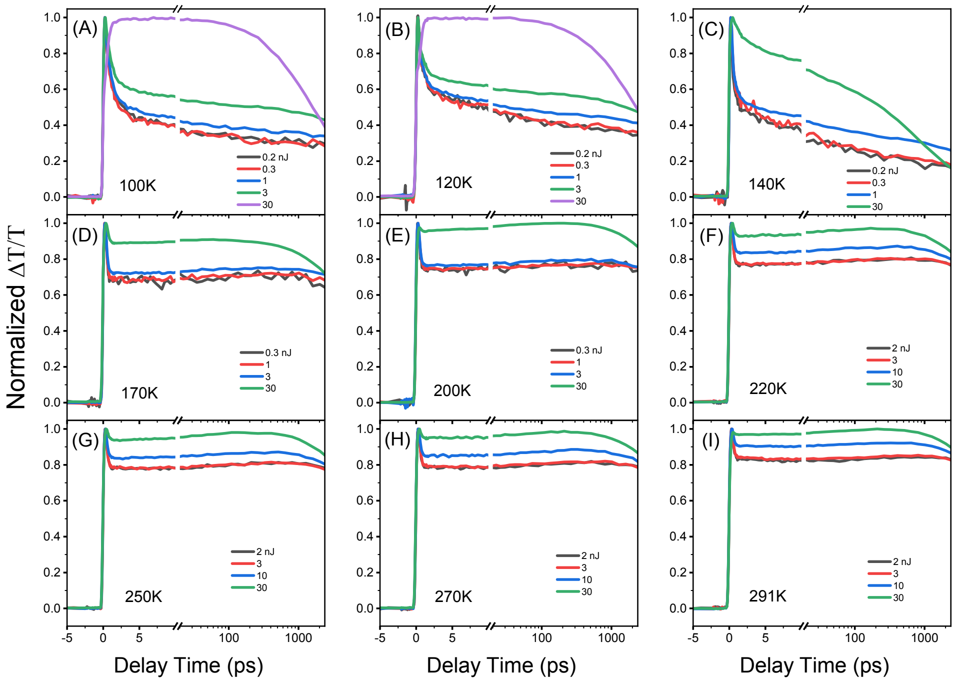

Figure S4. Carrier density-dependent TA dynamics excited by femtosecond laser pulses at different temperatures. A 3 mm diameter femtosecond laser with a wavelength of 517.5 nm was used as the pump light. The wavelengths of the bleaching peaks at each temperature obtained from Figure S3 were used as the wavelengths of the probe light (diameter ~0.8 mm) at the corresponding temperatures, respectively.

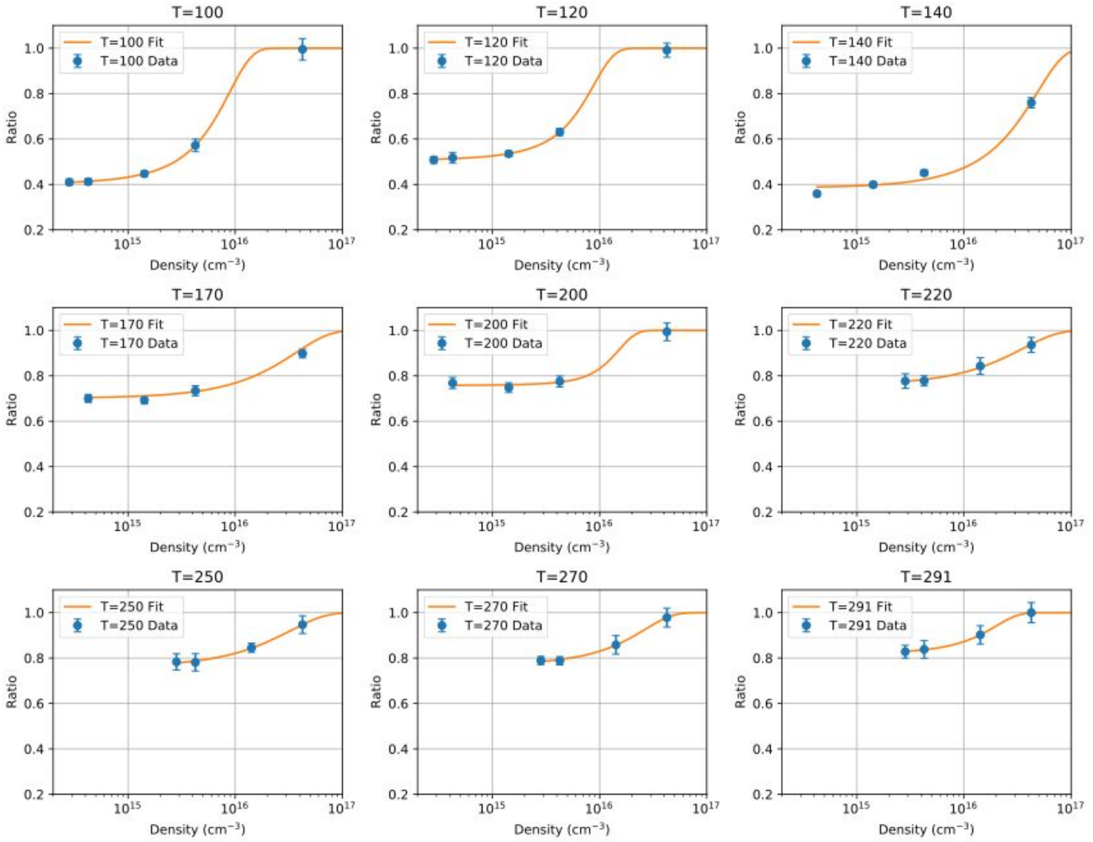

Figure S5. *B*/*A* ratio (blue datapoints) versus carrier density at all measured temperatures. The yellow lines are fits to an error function.

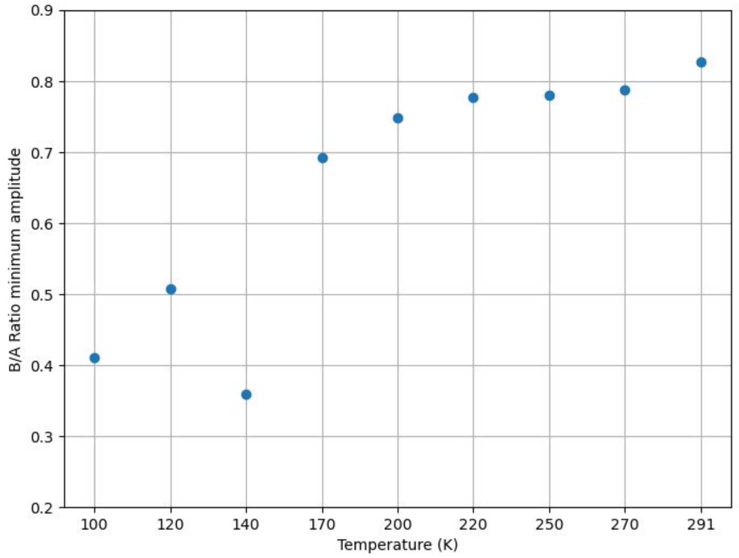

Figure S6. Amplitude of the *B*/*A* ratio error function fit versus temperature. Lower numbers in this case mean larger amplitudes. The deviation at 140K is likely due to the presence of both orthorhombic and tetragonal crystal phases.

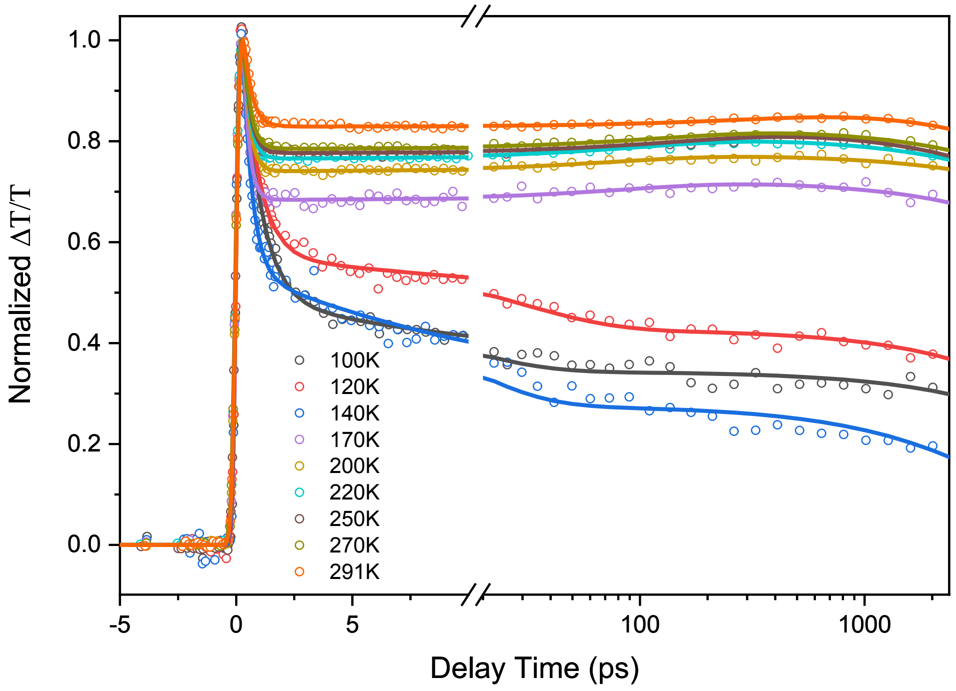

Figure S7: Comparison of TA dynamics excited by femtosecond laser at different temperatures in the linear response range. Dots are original data, and lines are fitting results.

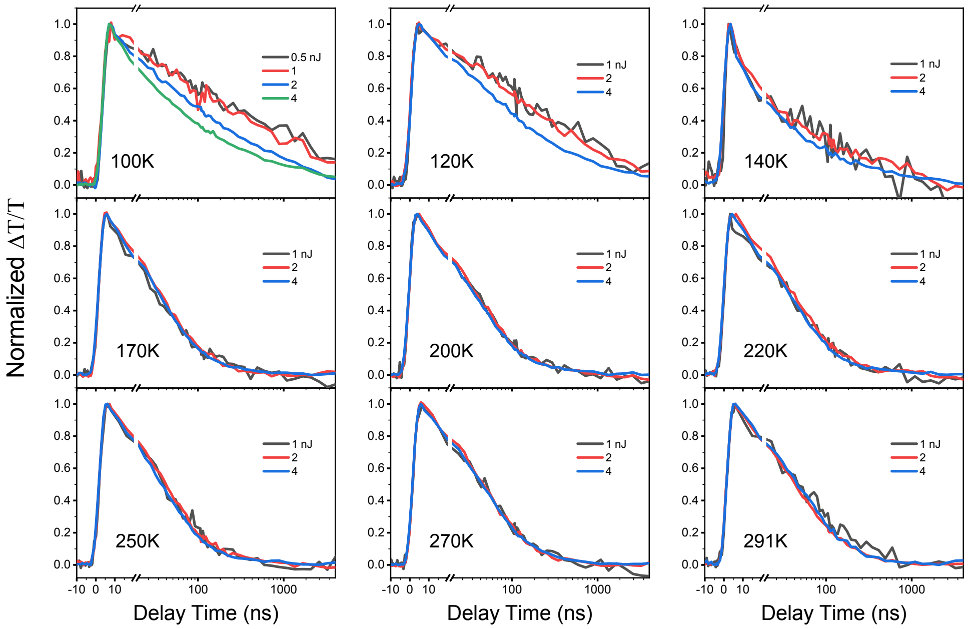

Figure S8: Carrier density-dependent TA dynamics excited by nanosecond laser at different temperatures. The pump light is a nanosecond laser with a spot size of about 8×2.5 mm^2. The wavelengths

of the bleaching peaks at each temperature obtained from Figure S3 were used as the wavelengths of the probe light (light spot diameter about 0.8 mm) at the corresponding temperatures, respectively.

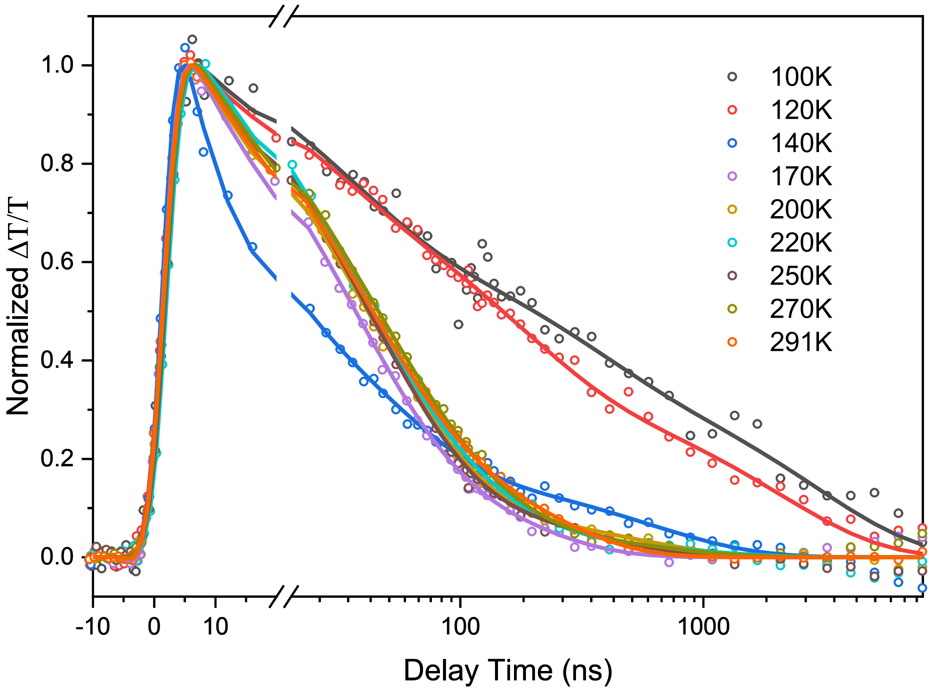

Figure S9: Comparison of TA dynamics excited by nanosecond laser at different temperatures in the linear response range. Dots are original data, and lines are fitting results.

Temp. (K)	Res. (ps)	A_1	t_1 (ps)	A_2	t_2 (ps)	A_3	t_3 (ns)
100	0.28 ±0.00	0.690 ±0.014	0.796 ±0.034	0.143 ±0.001	13.5 ±1.8	0.330 ±0.005	17.1 ±3.3
120	0.27 ±0.01	0.609 ±0.017	0.718 ±0.035	0.144 ±0.001	28.3 ±4.2	0.416 ±0.001	16.4 ±3.1
140	0.30 ±0.01	0.830 ±0.044	0.411 ±0.034	0.265 ±0.012	13.2 ±1.2	0.270 ±0.008	5.2 ±0.8
170	0.36 ±0.01	0.949 ±0.053	0.242 ±0.013	-0.039 ±0.005	95.8 ±34.2	0.715 ±0.006	38.2 ±6.9
200	0.36 ±0.01	0.752 ±0.033	0.263 ±0.011	-0.033 ±0.003	84.0 ±23.7	0.763 ±0.004	62.3 ±11.5
220	0.36 ±0.00	0.699 ±0.029	0.265 ±0.010	-0.043 ±0.003	110.2 ±23.2	0.804 ±0.004	42.4 ±4.9
250	0.36 ±0.00	0.646 ±0.029	0.278 ±0.011	-0.046 ±0.004	171.4 ±35.9	0.823 ±0.005	37.1 ±4.2
270	0.37 ±0.00	0.625 ±0.026	0.284 ±0.011	-0.041 ±0.004	144.7 ±31.9	0.824 ±0.004	44.1 ±5.4
291	0.36 ±0.00	0.448 ±0.021	0.334 ±0.015	-0.039 ±0.010	418.1 ±168.0	0.867 ±0.011	47.3 ±11.8

Table S2. Fitting results of TA curve in Figure S7 with triple exponential function. A_1, A_2 and A_3 are the initial quantities of three decays, t_1, t_2 and t_3 are their lifetimes, and Res. is the resolution of TA system obtained from fitting. The data at 140 K are measured at the peak of 735 nm.

Full OPTP dataset and analysis

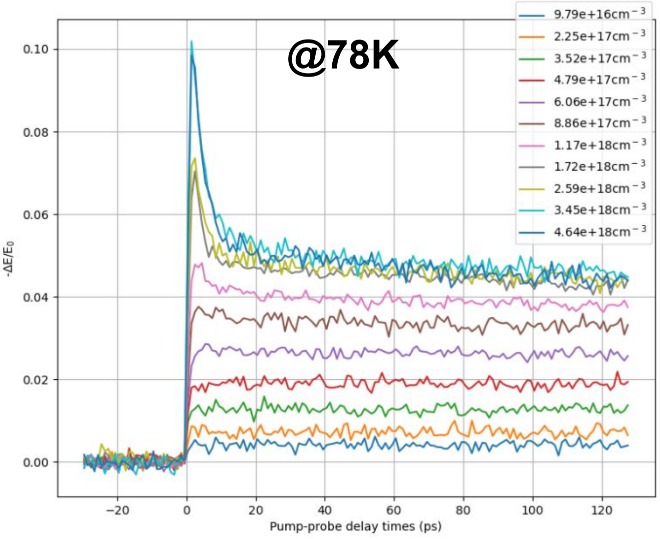

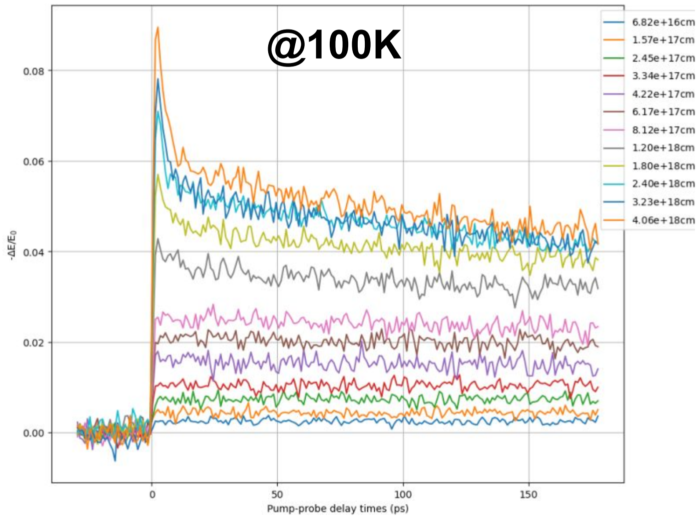

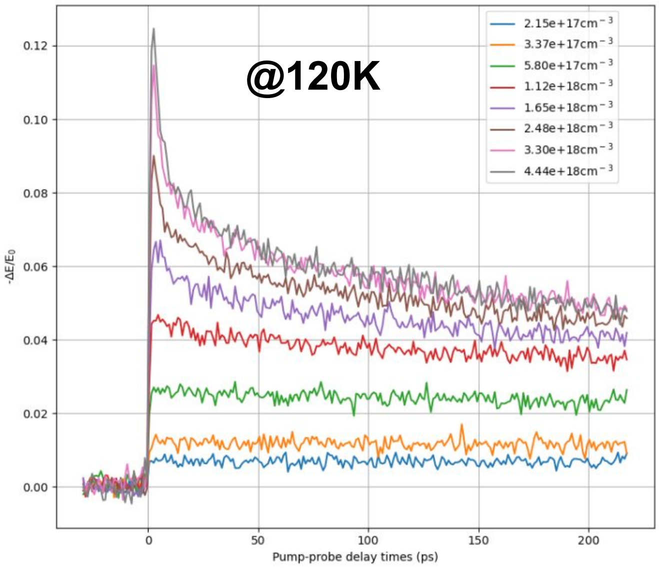

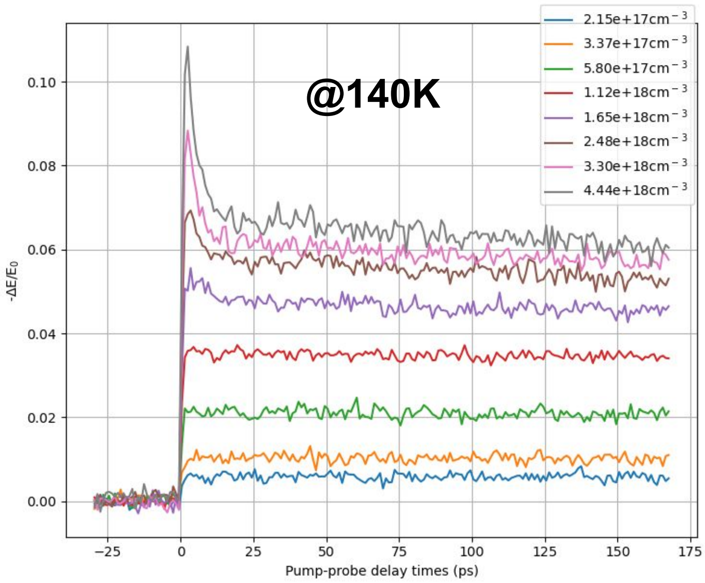

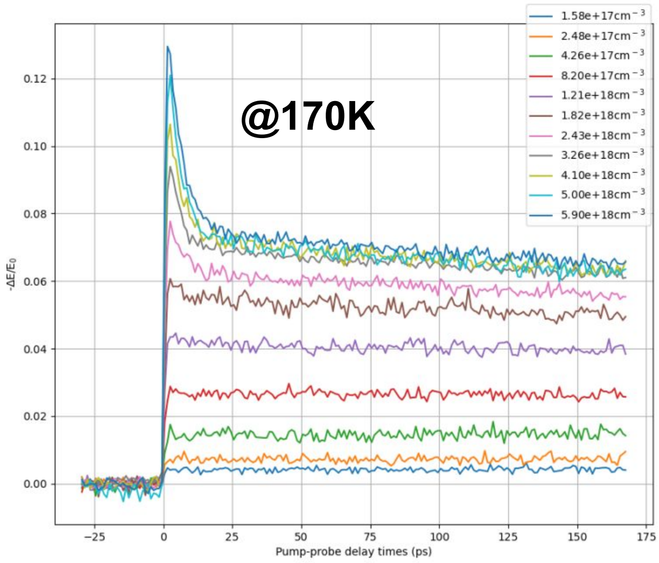

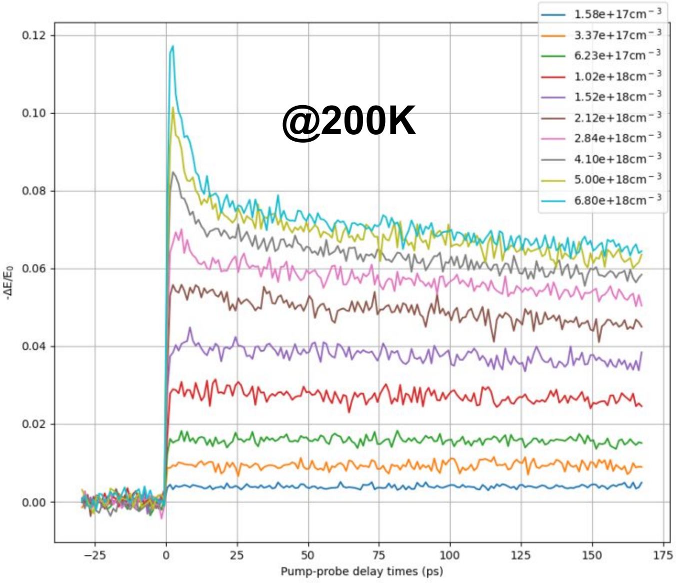

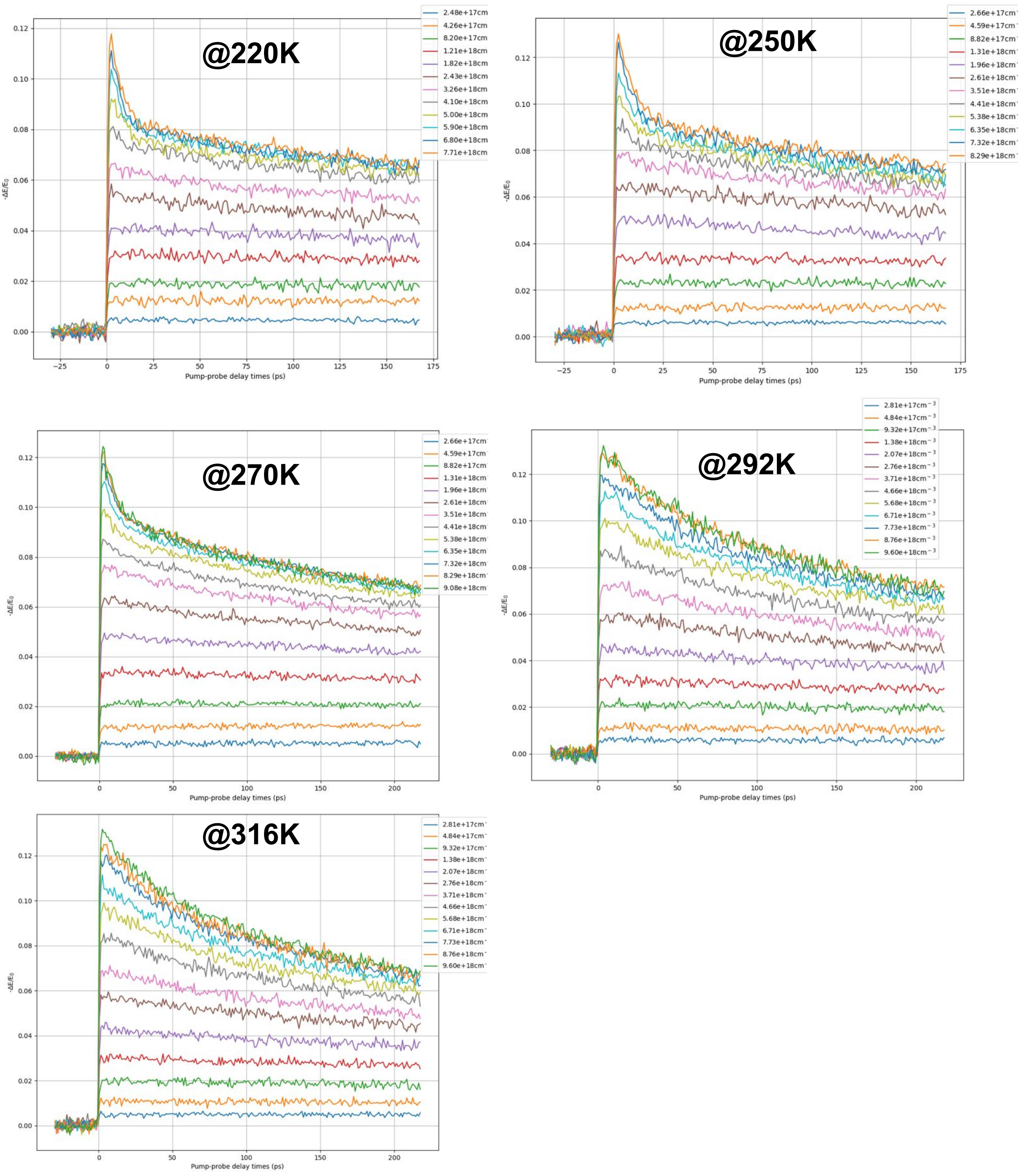

Figure S10: Real part of the OPTP signal, by measuring the photoinduced change in the transmitted THz field -$\Delta E/E_0$ (proportional to the photoconductivity) at the peak of the THz waveform. Photoexcited carrier densities are indicated in the panel.

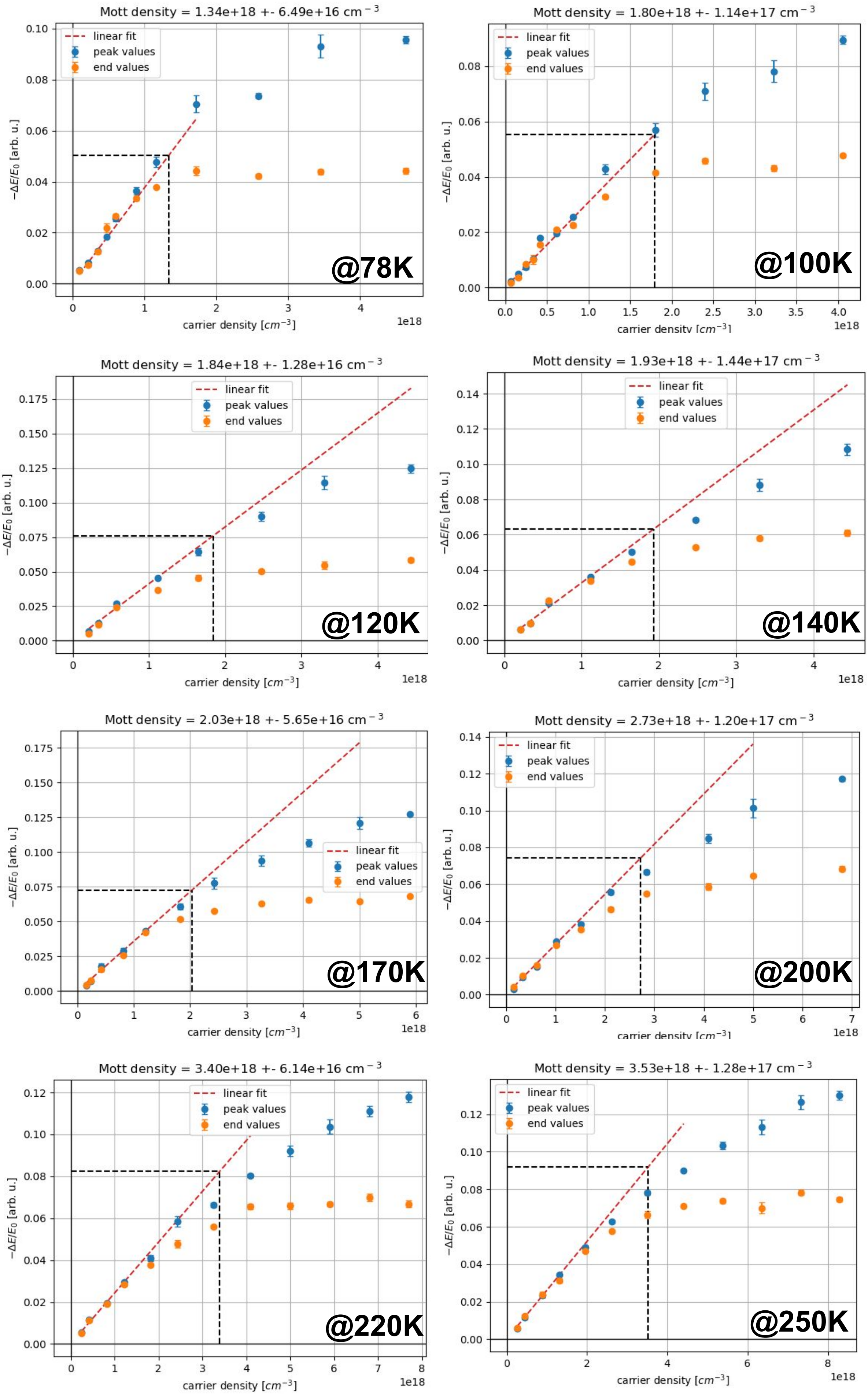
Mott density = 1.34e+18 +- 6.49e+16 cm⁻³
@78K
Mott density = 1.80e+18 +- 1.14e+17 cm⁻³
@100K
Mott density = 1.84e+18 +- 1.28e+16 cm⁻³
@120K
Mott density = 1.93e+18 +- 1.44e+17 cm⁻³
@140K
Mott density = 2.03e+18 +- 5.65e+16 cm⁻³
@170K
Mott density = 2.73e+18 +- 1.20e+17 cm⁻³
@200K
Mott density = 3.40e+18 +- 6.14e+16 cm⁻³
@220K
Mott density = 3.53e+18 +- 1.28e+17 cm⁻³
@250K
linear fit
peak values
end values
−ΔE/E0 [arb. u.]
carrier density [cm⁻³]
1e18

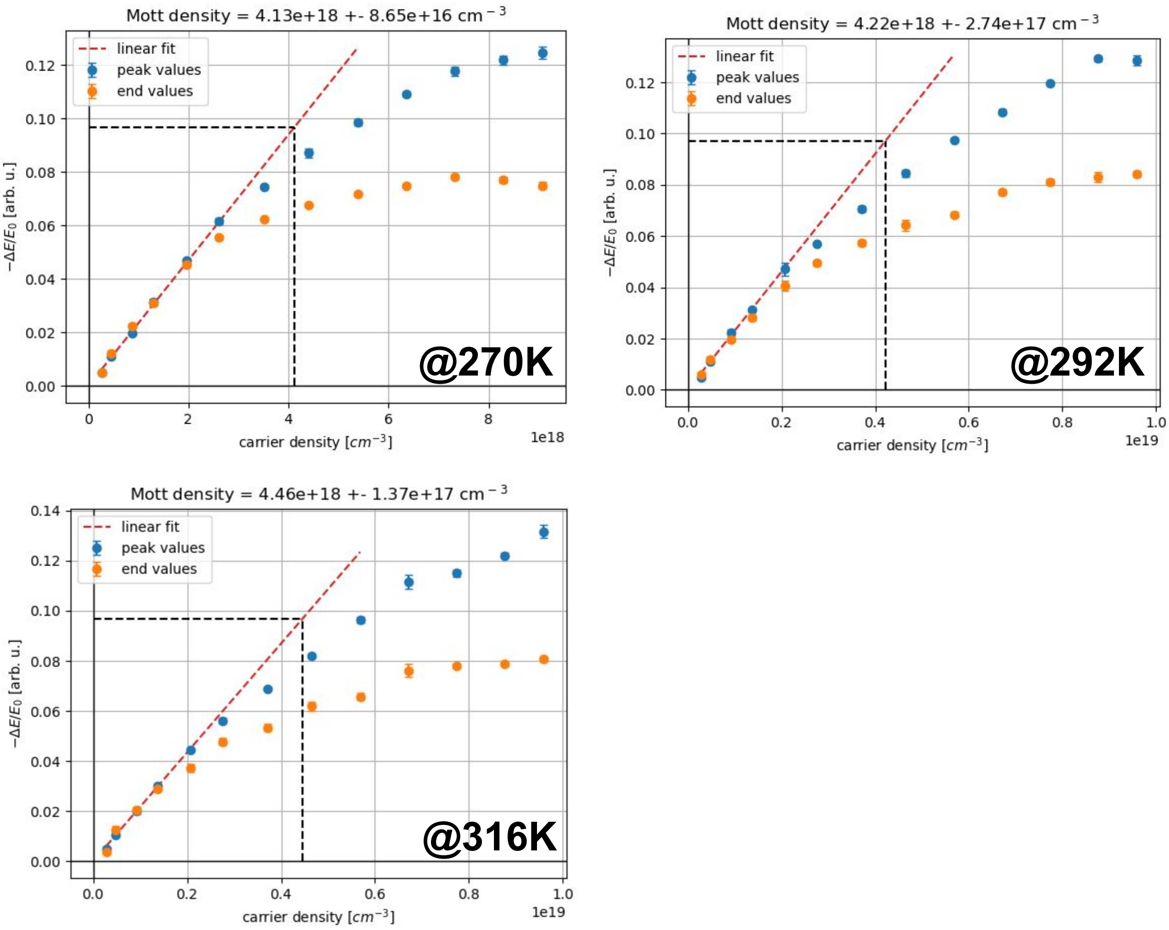

Figure S11: Determination of the Mott density from the real part of the OPTP transients at various temperatures, acquired from the data shown in Figure SXX. Blue datapoints are the -$\Delta E/E_0$ values at the peak of the OPTP signal (averaged from 0.5-1.5 ps pump-probe delay time). The yellow datapoints are the average -$\Delta E/E_0$ values over the last 10 ps of the OPTP transients. Error bars indicate standard deviations over the averaged time window. The red dashed line is a linear fit to the lowest three to five datapoints of the peak -$\Delta E/E_0$ value, where we have selected the range over which the increase of the signal was linear with increasing carrier density (i.e. pump fluence). We used the linear fit, as explained throughout the main text, to determine the Mott density at each temperature, which is indicated above each panel.

Estimation of the temperature-dependent polaron size

At the Mott density, the polaron wavefunctions in MAPI start overlapping and annihilate rapidly. Assuming spherical wavefunctions, which occupy in a most-dense configuration where 74% of space is filled, we can state that the ratio of all volume occupied by polarons w.r.t. the total volume follows:

$$\frac{\#polarons \cdot \frac{4}{3}\pi r_{polaron}^3}{V} = 0.74$$

Where $r_{polaron}^3$ is the radius of one polaron cubed. We then use the definition of the Mott density N_{mott} to calculate the radius of one polaron, which is converted into a diameter in Figure S5 below:

$$N_{mott} \cdot \frac{4}{3}\pi r_{polaron}^3 = 0.74$$

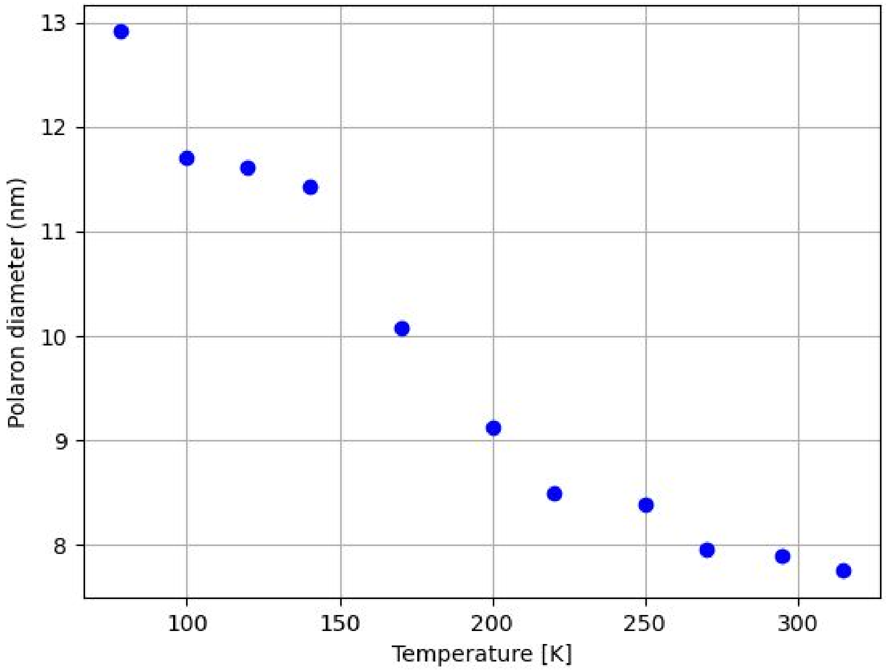

Figure S12: Polaron diameter as a function of temperature, as obtained from the experimental OPTP data.

As can be seen, the polaron diameter increases with decreasing temperature. Moreover, we confirm that the polaron wavefunctions span several unit cells (lattice parameters ~0.6 nm), showing that large polarons are formed in MAPI over the entire temperature range we probed experimentally.

Modeling the threshold for optical gain/population inversion

We follow the same approach as Suárez et al.[3], which model at which density MAPI becomes transparent and shows optical gain, i.e. the density at which there is population inversion[4,5]. They follow the classic Bernard-Duraffourg conditions for lasing, namely that the splitting of quasi-Fermi levels (QFL) should be larger than or equal to the bandgap energy:

$$Ef_c - E_{f,v} \geq E_g$$

Or equivalently

$$Ef_c - E_{f,v} - E_g = 0$$

For this, we need to calculate how much a photoexcited electron and hole raise or lower the QFL towards the conduction band (for electrons) and valence band (for holes), respectively, for which we need to know the density of states. We use the density of a free electron gas in three dimensions in a parabolic band with effect mass m^*:

$$N(E) = \int_{E_c}^{E} dE \, \frac{1}{2\pi^2}\left(\frac{2m_e^*}{\hbar^2}\right)^{3/2} \sqrt{E} \, \frac{1}{1 + e^{\frac{E_c - E_{f,c}}{k_B T}}}$$

With m_e^* the effective mass of the first conduction band, E_c the energy of the conduction band minimum, $E_{f,c}$ the QFL of the electrons and $N(E)$ the density of states per unit volume. The latter term in the integral is the Fermi-Dirac distribution for the electrons.

A similar equation is set up for the holes. We calculate how much the addition of one electron (hole) raises (lowers) the QFL for the electrons.

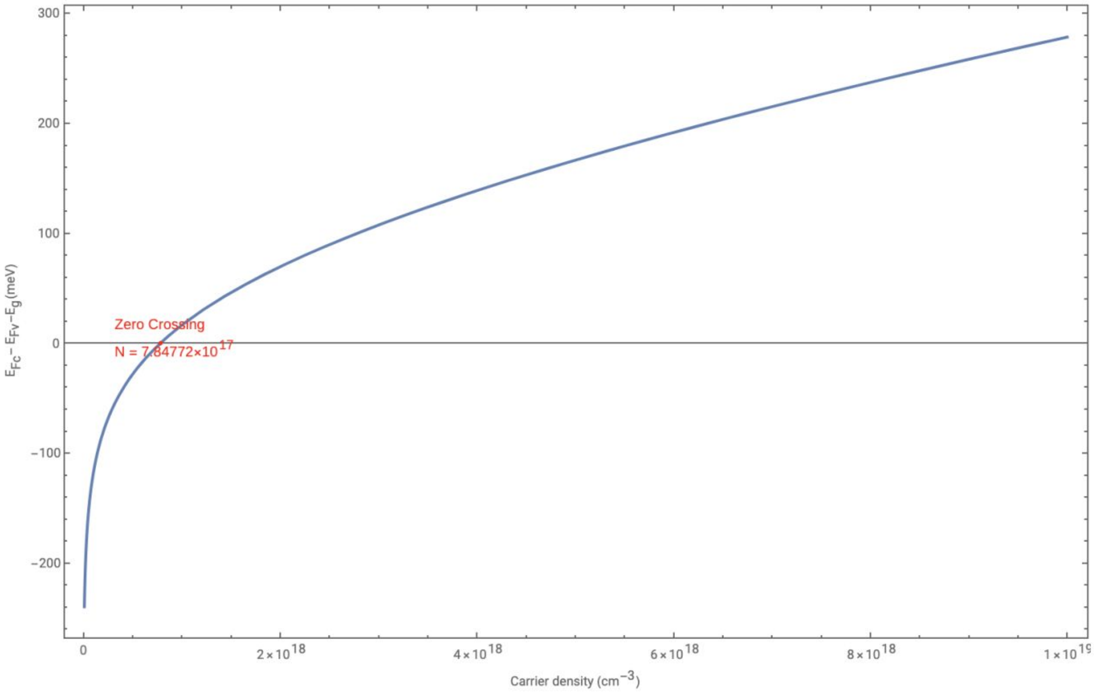

Figure S13: example calculations of the threshold for optical gain in MAPI, with m_e^*,m_h^* = 0.11, 0.13. We have changed both temperature and the electron and hole effective masses to obtain the temperature-and-density-dependent curves shown in Figure 3 of the main text.

We repeated the calculations to determine the density for population inversion for a range of temperatures, the results of which are shown in Figure 3 of the main text. We benchmarked the code and obtained the same results as Suárez et al.[3] for both MAPI and GaAs. Note that we do not consider excitonic effects, or fast-and-strong electron-phonon interactions, which would reduce the threshold for optical gain since the stimulated emission band gets redshifted out of the spectral range in which MAPI absorbs light.

Supplementary references

(1) Leguy, A. M. A.; Hu, Y.; Campoy-Quiles, M.; Alonso, M. I.; Weber, O. J.; Azarhoosh, P.; van Schilfgaarde, M.; Weller, M. T.; Bein, T.; Nelson, J.; Docampo, P.; Barnes, P. R. F. Reversible Hydration of CH3NH3PbI3 in Films, Single Crystals, and Solar Cells. *Chem. Mater.* **2015**, *27* (9), 3397–3407. https://doi.org/10.1021/acs.chemmater.5b00660.

(2) Gao, L.; Zhang, H.; Zhang, Y.; Fu, S.; Geuchies, J. J.; Valli, D.; Saha, R. A.; Pradhan, B.; Roeffaers, M.; Debroye, E.; Hofkens, J.; Lu, J.; Ni, Z.; Wang, H. I.; Bonn, M. Tailoring Polaron Dimensions in Lead-Tin Hybrid Perovskites. *Advanced Materials n/a* (n/a), 2406109. https://doi.org/10.1002/adma.202406109.

(3) Suárez, I.; Juárez-Pérez, E. J.; Chirvony, V. S.; Mora-Seró, I.; Martínez-Pastor, J. P. Mechanisms of Spontaneous and Amplified Spontaneous Emission in ${\mathrm{CH}}_{3}{\mathrm{NH}}_{3}{\mathrm{Pb}\mathrm{I}}_{3}$ Per ovskite Thin Films Integrated in an Optical Waveguide. *Phys. Rev. Appl.* **2020**, *13* (6), 064071. https://doi.org/10.1103/PhysRevApplied.13.064071.

(4) Bernard, M. G. A.; Duraffourg, G. Laser Conditions in Semiconductors. *physica status solidi (b)* **1961**, *1* (7), 699–703. https://doi.org/10.1002/pssb.19610010703.

(5) Rosencher, E.; Vinter, B. *Optoelectronics*; Piva, P. G., Translator; Cambridge University Press: Cambridge, 2002. https://doi.org/10.1017/CBO9780511754647.
