# Supplementary material for: Temperature-dependent trapping and polaron annihilation on ultrafast time scales in metal-halide perovskites
Source: J Phys Chem Lett. 2025 Sep 12;16(38):9925–32. doi: 10.1021/acs.jpclett.5c02164 (PMC12478862; doi:10.1021/acs.jpclett.5c02164)
Supplement: Supplementary file 2 [file jz5c02164_si_002.pdf]

jz-2025-021646.R1

Name: Peer Review Information for "Temperature-dependent trapping and polaron annihilation on ultrafast timescales in metal-halide perovskites"

First Round of Reviewer Comments

Reviewer: 1

Comments to the Author

The manuscript by Wang and co-workers reports data on the temperature- and carrier-density dependent electronic properties of the prototypical halide perovskite MAPI, using transient absorption and optical-pump THz probe spectroscopy.

While I must emphasize that neither the sample and material investigated nor the techniques employed to do so or the major conclusions drawn from the experiments are inherently novel, the quality of the data presented is very high. As such, despite absence of novelty, I would recommend publication of these data sets for the community.

My only concern is with the TA data fitting, where simply triexponential decays were fitted to the kinetics (SI, Table S2 reports some of them). Considering the last 10-15 years of spectroscopy carried out on MAPI, the community here really has progressed beyond such a phenomenological fit and a more adequate drift-diffusion-recombination model should be expected to be employed (see e.g. nice recent works by Thomas Kirchartz on how to fit recombination kinetics, or earlier works by Sum or Herz).

Once the fits are revised and proper recombination constants for mono-, bi- and tri-molecular (Auger) recombination of non-radiative and radiative nature including surface velocities are analysed, I would recommend publication of this work in JPCL.

Reviewer: 2

Comments to the Author

I previously reviewed this manuscript when it was submitted to J. Am. Chem Soc. My assessment of the work remains broadly positive: the topic is timely, the methodology is sound, and the results are of interest to the community. However, I note that none of the suggestions I made in my prior review appear to have been addressed in this resubmission.

I still recommend publication after revision, but I urge the authors to carefully consider and respond to the previously raised points, which remain relevant. For clarity, I am including my earlier review below.

This paper presents photoconductivity measurements of methylammonium lead iodide (MAPI) using optical-pump/THz-probe (OPTP) and transient absorption spectroscopy (TAS). The photoresponse of MAPI has been extensively studied due to its relevance for optoelectronic applications. However, this study makes a significant contribution by providing consistent data across a wide range of photoexcited carrier densities (10^14–10^19 cm^-3). This allows the authors to extract meaningful thresholds (an “electronic phase diagram”), including an estimate of the carrier density required for population inversion, highlighting the potential of MAPI for lasing applications. On this basis, I believe the paper may be suitable for publication, provided the authors address the following points:

1. The authors describe the Mott transition threshold as a material-inherent property, in contrast to the trap density, which is sample-dependent. While I agree that the underlying mechanism for the Mott transition, i.e., many-body screening and polaron overlap, is governed by intrinsic material properties, I wonder whether, in real samples with significant and possibly inhomogeneous trap densities, the apparent threshold for such a transition could be affected by local screening environments. In that case, the observed crossover density may not be entirely independent of synthesis conditions. Some discussion of this potential sample-to-sample variability might be helpful for readers.

2. In the high carrier density regime, the authors attribute the ultrafast carrier decay to an "Auger-type" recombination process. While this assignment is plausible and consistent with the experimental data, the authors should also discuss possible alternative explanations. As far as I can see, no direct evidence for Auger recombination is presented, and the observed dynamics could in principle also arise from density-dependent trapping or mobility reduction due to many-body scattering. It would strengthen the paper if the authors either provided more justification for this interpretation or more explicitly framed it as a working hypothesis rather than a definitive assignment.

3. Finally I am missing a discussion of the potential role of excitonic effects at low temperatures and low excitation densities. While the authors neglect excitons throughout their analysis, which I think is justified at room temperature and in the high-density regime, it is less clear whether this assumption holds in the orthorhombic phase at low temperature. Given the exciton binding energy in MAPI (~16 meV), exciton formation could influence both the TAS and the carrier dynamics under those conditions.

Author's Response to Peer Review Comments:

Dear editor,

We thank you for sharing the feedback from the reviewers with us. Overall, we are positive and very thankful for the feedback from the reviewers and feel confident that upon resubmission our works is ready to be published. In the rebuttal document, please find this attached, we answer the questions and comments from both reviewers and highlight where actions are taken.

Please not that we have changed the title of our manuscript, as to better cover the content: Temperature-dependent trapping and polaron annihilation on ultrafast timescales in metal-halide perovskites

Sincerely, no behalf of all authors,

Jaco Geuchies

Rebuttal JPCL

We thank the editor for sharing the feedback from the reviewers with us. Overall, we are positive and thankful for the feedback from the reviewers and feel confident that upon resubmission our works is ready to be published. Below we answer the questions and comments from both reviewers and highlight where actions are taken.

Comments from the reviewer are given in between quotation marks "...", and our answers are stated directly below in italic font.

Reviewer 1

"Recommendation: This paper may be publishable, but major revision is needed; I would like to be invited to review any future revision."

Answer: We thank the reviewer for their constructive feedback.

"Comments:
The manuscript by Wang and co-workers reports data on the temperature- and carrier-density dependent electronic properties of the prototypical halide perovskite MAPI, using transient absorption and optical-pump THz probe spectroscopy.

While I must emphasize that neither the sample and material investigated nor the techniques employed to do so or the major conclusions drawn from the experiments are inherently novel, the quality of the data presented is very high. As such, despite absence of novelty, I would recommend publication of these data sets for the community. "

Answer: We thank the reviewer for their positive feedback. We would like to emphasize that an important message for the community concerns the importance of carefully measuring the densities at which ultrafast experiments are performed. Reporting fluence on its own is not enough: the quantity that matters for the dynamics in the excited state is the photogenerated carrier density. As shown in our work, trapping at low densities and fast polaron-polaron annihilation occur on similar timescales, but have opposite dependencies on pump-fluence (i.e. density). This is not only relevant for perovskite-based materials, but for all ultrafast spectroscopic

experiments on (semiconductor and quantum) materials. Note that especially the ultralow fluence TAS studies are in a density regime that is not extensively studied due to experimental difficulty measuring weak signals at these low densities.
Action: we emphasize the importance of our work by slightly rephrasing the title of our paper, which now reads "Temperature-dependent trapping and polaron annihilation on ultrafast timescales in metal-halide perovskites".

"My only concern is with the TA data fitting, where simply triexponential decays were fitted to the kinetics (SI, Table S2 reports some of them). Considering the last 10-15 years of spectroscopy carried out on MAPI, the community here really has progressed beyond such a phenomenological fit and a more adequate drift-diffusion-recombination model should be expected to be employed (see e.g. nice recent works by Thomas Kirchartz on how to fit recombination kinetics, or earlier works by Sum or Herz).

Once the fits are revised and proper recombination constants for mono-, bi- and trimolecular (Auger) recombination of non-radiative and radiative nature including surface velocities are analysed, I would recommend publication of this work in JPCL."

Answer: We sincerely appreciate the practical suggestions from the reviewer regarding the method for fitting transient absorption data in our manuscript. We already reviewed and cited the articles by Thomas Kirchartz et al. (e.g. Shallow Defects and Variable Photoluminescence Decay Times up to 280 ms in Triple-Cation Perovskites, doi: 10.1038/s41563-023-01771-2*) in our work. The fitting of driftdiffusion-recombination models have provided inspiration for improving our future research.*

The TA curves in this work reflect the carrier dynamics at very low carrier densities. In this manuscript, the focus for the TA spectroscopy we carried out was to study dynamics under low carrier density and, importantly, at short (sub-100 ps) pump-probe delay times. At low carrier densities (the “linear response range” as described in the manuscript), the carrier dynamics are independent of the carrier density. Monomolecular recombination is the main decay process, and bi-molecular and trimolecular recombination (Auger recombination only occurs at much higher densities, as shown in the OPTP experiments) processes can be ignored at early times and these low densities. The carrier dynamics in the linear response range

reflect the true carrier dynamics of solar cell materials under photon fluxes equivalent to the intensity of one sun.

Concerning the charge carrier drift and diffusion the reviewer points out, these processes do not show up in our TA curves under low carrier density. The pump light diameter is ~3mm, and the probe light diameter is ~0.8 mm. On the timescale of our experiments, lateral diffusion therefore does not reach these length scales, and as such, our setup is insensitive to these effects. In addition, longitudinal diffusion, perpendicular to the film surface, cannot be captured by our setup. Hence, drift and diffusion are not captured in our technique and in the resulting data analysis. For future studies, we are currently developing a high-sensitivity transient absorption microscope, which we will use to study drift and diffusion processes of solar cell materials.

Action: As stated earlier, we emphasize the importance of our work by slightly rephrasing the title of our paper, which now reads "Temperature-dependent trapping and polaron annihilation on ultrafast timescales in metal-halide perovskites".

Additionally, we have added a sentence to further clarify this on page. 10, where we cite the work from the Kirchartz group again:

“Note that in our TAS experiments, we use pump and probe lateral sizes of three and one mm, respectively (see experimental methods). Effects such as drift and diffusion do not occur over these length- and time scales[29]. Factors affecting carrier dynamics, in addition to crystal structure, may also include the energy distribution and spatial distribution of shallow- and deep-level defect states."

Reviewer: 2

Recommendation: This paper is publishable subject to minor revisions noted. Further review is not needed.

Answer: We thank the reviewer for their positive assessment of our work.

"Comments:
I previously reviewed this manuscript when it was submitted to J. Am. Chem Soc. My assessment of the work remains broadly positive: the topic is timely, the methodology is sound, and the results are of interest to the community. However, I note that none of the suggestions I made in my prior review appear to have been addressed in this resubmission.

I still recommend publication after revision, but I urge the authors to carefully consider and respond to the previously raised points, which remain relevant. For clarity, I am including my earlier review below.

This paper presents photoconductivity measurements of methylammonium lead iodide (MAPI) using optical-pump/THz-probe (OPTP) and transient absorption spectroscopy (TAS).
The photoresponse of MAPI has been extensively studied due to its relevance for optoelectronic applications. However, this study makes a significant contribution by providing consistent data across a wide range of photoexcited carrier densities (10^14–10^19 cm^-3). This allows the authors to extract meaningful thresholds (an "electronic phase diagram"), including an estimate of the carrier density required for population inversion, highlighting the potential of MAPI for lasing applications. On this basis, I believe the paper may be suitable for publication, provided the authors address the following points:"

Answer: We thank the reviewer for their positive assessment of our work and give a point-by-point answer to their feedback below.

"1. The authors describe the Mott transition threshold as a material-inherent property, in contrast to the trap density, which is sample-dependent. While I agree that the underlying mechanism for the Mott transition, i.e., many-body screening and polaron overlap, is governed by intrinsic material properties, I wonder whether, in real samples with significant and possibly inhomogeneous trap densities, the apparent threshold for such a transition could be affected by local screening environments. In that case, the observed crossover density may not be entirely independent of synthesis conditions. Some discussion of this potential sample-to-sample variability might be helpful for readers."

Answer: The reviewer points out that there might be sample-to-sample variations in the measured Mott density due to differences in trap densities and inhomogeneities therein. While we agree there may be small differences, due to local changes in

screening, overall, the Mott density follows the prediction made by Feynmann's polaron model, as we have shown in previous work (https://doi.org/10.1021/acsenergylett.2c01949, ACS Energy Lett. 2023, 8,420−428; https://advanced.onlinelibrary.wiley.com/doi/full/10.1002/adma.202406109, Advanced Materials, 2024, 2406109*). Furthermore, the Mott densities (around 10^{18} cm^{-3}) are two orders of magnitude above shallow trap densities, and six orders of magnitude above deep trap densities. This mismatch implies that variations of trap densities is unlikely to substantially affect the crossover Mott density. Careful validation of this statement with various samples has been done recently by us in different sets of MAPI samples (https://advanced.onlinelibrary.wiley.com/doi/full/10.1002/adma.202406109,* Advanced Materials, 2024, 2406109*).*

"2. In the high carrier density regime, the authors attribute the ultrafast carrier decay to an "Auger-type" recombination process. While this assignment is plausible and consistent with the experimental data, the authors should also discuss possible alternative explanations. As far as I can see, no direct evidence for Auger recombination is presented, and the observed dynamics could in principle also arise from density-dependent trapping or mobility reduction due to many-body scattering. It would strengthen the paper if the authors either provided more justification for this interpretation or more explicitly framed it as a working hypothesis rather than a definitive assignment. "

Answer: The reviewer correctly points out that we measure photoconductivity, i.e. a product of carrier density times mobility (times a quantum yield) in our OPTP experiments. A reduction of the OPTP signal can therefore come from a reduction in mobility or a loss of carrier density (e.g. due to trapping).

We argue that the loss in photoconductivity does not originate from density-dependent trapping: at the crossover density, we are two orders above the shallow trap density, and six orders of magnitude above the deep trap density, meaning that all possible trap sites should be saturated, which we also prove from the saturating TAS signal at low-to-intermediate densities and the saturating OPTP signals at intermediate-to-high densities.

Mobility reduction due to many-body scattering occurs simultaneously with a loss in carrier density, which we have shown in earlier work (https://doi.org/10.1021/acsenergylett.2c01949 ACS Energy Lett. 2023, 8,420−428). We agree that the statement concerning Auger recombination can be rephrased better as a working hypothesis. Additionally, this claim can be verified by performing TAS experiments at high densities as well, since the signal is mostly sensitive to changes in the population of photoexcited carriers, and to a lesser degree to a loss in carrier mobility.

Action: We have toned down the claim that the polaron annihilation process and rephrased the sentence in the abstract as:

" overlapping polaron wavefunctions lead to ultrafast annihilation, tentatively assigned as an Auger recombination process, occurring over a few picoseconds".

on page 12 as:

"likely via an Auger-type mechanism"

and on page 13, we removed the mention of Auger recombination, and simply rephrased as:

" This is illustrated by the similar transient signatures of polaron-polaron annihilation at high densities."

"3. Finally I am missing a discussion of the potential role of excitonic effects at low temperatures and low excitation densities. While the authors neglect excitons throughout their analysis, which I think is justified at room temperature and in the high-density regime, it is less clear whether this assumption holds in the orthorhombic phase at low temperature. Given the exciton binding energy in MAPI (~16 meV), exciton formation could influence both the TAS and the carrier dynamics under those conditions. "

Answer: The reviewer rightfully points out the presence of possible excitonic effects at low temperatures and carrier densities. Excitonic effects are often congested in THzphotoconductivity spectra, especially in samples with low exciton binding

energies. Pure excitonic responses are characterized by an imaginary photoconductivity that is negative and increases with initial frequency (into a Gaussian-derivative-like shape), and a real photoconductivity that increases as a function of frequency, described by a

Lorentzian oscillator. Other very insightful studies, e.g., on FAPI by the Herz group (https://doi.org/10.1021/acs.jpclett.8b01628), have shown that excitonic effects only play a minor role, even at temperatures as low as 10 K. Clear and clean excitonic signatures in photoconductivity spectra have, to the best of our knowledge, only been seen in systems with very high exciton binding energies, such as graphene nanoribbons (binding energy of 700 meV, https://doi.org/10.1021/acs.nanolett.9b04816) and ZnO (https://journals.aps.org/prb/abstract/10.1103/PhysRevB.76.045214)

Another piece of experimental evidence pointing to the negligible role of excitons in our study is the temperature dependence of the quantum yield (QY). One of the consequences of exciton formation is a less-than-unity photon-to-mobile-charge QY. As we have shown in our supplementary information, we extract a QY of 55% at 78K, and a QY of 30% at 292K. We do not go into the detailed interpretation of these numbers, as any loss of carriers will contribute to this number, not just exciton formation. It is, however, noteworthy that the QY increases with decreasing temperature, which goes against the hypothesis that at lower temperatures more excitons form (theoretically leading to a decrease in the QY). In our opinion, a thorough understanding of the photoconductivity spectra in perovskite materials is key to making any conclusive statements on the exact nature of the QY as a function of temperature, which is part of ongoing work and goes beyond the message of our current paper.

Action: we have added the reference to the work from the Herz group above to our references, and added the following sentence to the main text (page 6) when discussing the measured QYs:

'Less than unity quantum-yield in these materials is amongst others linked to exciton formation[41].'

jz-2025-021646.R2

Name: Peer Review Information for "Temperature-dependent trapping and polaron annihilation on ultrafast timescales in metal-halide perovskites"

Second Round of Reviewer Comments

Reviewer: 1

Comments to the Author

I am mostly satisfied with the authors' responses and revisions and can recommend publication.

Author's Response to Peer Review Comments:

Dear Editor,

Thank you for the very positive news. We are very grateful to publish this work in JPCL. Attached you can find the updated manuscript, in which we have moved the email addresses of all corresponding authors to the first page, as requested.

All my best, on behalf of all co-authors,

Jaco Geuchies
